# SERS Based Lateral Flow Assay for Rapid and Ultrasensitive Quantification of Dual Laryngeal Squamous Cell Carcinoma-Related miRNA Biomarkers in Human Serum Using Pd-Au Core-Shell Nanorods and Catalytic Hairpin Assembly

**DOI:** 10.3389/fmolb.2021.813007

**Published:** 2022-02-11

**Authors:** Guang Li, Ping Niu, Shengjie Ge, Dawei Cao, Aidong Sun

**Affiliations:** ^1^ Department of Otorhinolaryngology-Head and Neck Surgery, The Affiliated Hospital of Yangzhou University, Yangzhou University, Yangzhou, China; ^2^ Departments of Otolaryngology, The Affiliated Hospital of Shandong First Medical University, Qingzhou People’s Hospital, Qingzhou, China; ^3^ Institute of Translational Medicine, Medical College, Yangzhou University, Yangzhou, China; ^4^ College of Mathematics and Computer Science, Zhejiang Normal University, Jinhua, China

**Keywords:** surface-enhanced Raman scattering, Pd-Au core-shell nanorods, lateral flow assay, catalytic hairpin assembly, miRNA, laryngeal squamous cell carcinoma

## Abstract

Non-invasive early diagnosis is of great significant in disease pathologic development and subsequent medical treatments, and microRNA (miRNA) detection has attracted critical attention in early cancer screening and diagnosis. However, it was still a challenge to report an accurate and sensitive method for the detection of miRNA during cancer development, especially in the presence of its analogs that produce intense background noise. Herein, we developed a surface-enhanced Raman scattering (SERS)–based lateral flow assay (LFA) biosensor, assisted with catalytic hairpin assembly (CHA) amplification strategy, for the dynamic monitoring of miR-106b and miR-196b, associated with laryngeal squamous cell carcinoma (LSCC). In the presence of target miRNAs, two hairpin DNAs could self-assemble into double-stranded DNA, exposing the biotin molecules modified on the surface of palladium (Pd)–gold (Au) core–shell nanorods (Pd-AuNRs). Then, the biotin molecules could be captured by the streptavidin (SA), which was fixed on the test lines (T1 line and T2 line) beforehand. The core–shell spatial structures and aggregation Pd-AuNRs generated abundant active “hot spots” on the T line, significantly amplifying the SERS signals. Using this strategy, the limits of detections were low to aM level, and the selectivity, reproducibility, and uniformity of the proposed SERS-LFA biosensor were satisfactory. Finally, this rapid analysis strategy was successfully applied to quantitatively detect the target miRNAs in clinical serum obtained from healthy subjects and patients with LSCC at different stages. The results were consistent with the quantitative real-time PCR (qRT-PCR). Thus, the CHA-assisted SERS-LFA biosensor would become a promising alternative tool for miRNAs detection, which showed a tremendous clinical application prospect in diagnosing LSCC.

## Introduction

Laryngeal squamous cell carcinoma (LSCC), a common clinical malignancy of the head and neck, is a type of tumor that arises mainly from the mucosal epithelium of the larynx, accounting for approximately 5% of systemic tumors and 15% of head and neck tumors worldwide (Steuer et al., 2017; Xu et al., 2020). The main reason for shallow low 5-year survival rate, less than 50% in advanced stages of the disease, is the late diagnosis (Gourin et al., 2009; Adam et al., 2012; Brandstorp-Boesen et al., 2017). Therefore, the early diagnosis of LSCC is vital for the targeted treatment. MicroRNAs (miRNAs), a newly discovered class of approximately 21- to 25-nt endogenous single-stranded nucleotide non-coding small RNA, play an essential role in various biological processes (Ferrua et al., 2019). Furthermore, the miRNAs expression level is closely associated with the occurrence and development of diseases, especially cancer, providing real-time information about the status of the disease; thus, rapid detection of miRNAs is of great significance for the evaluation of cancer progression (Aslam et al., 2009). As a possible cancer-promoting role in the progression of LSCC, miR-106b was significantly up-regulated in LSCC tissues (Yu et al., 2014). Many studies also showed that miR-196b targeted SOCS2 for regression and was prominently overexpressed in LSCC (Zhao et al., 2018). Thus, rapid and ultrasensitive of miR-106b and miR-196b could realize the dynamic monitoring of cancer development. Currently, numerous classical methods are employed to detect miRNAs, namely, real-time PCR (RT-PCR), digital PCR, and next-generation sequencing (NGS) (Yan et al., 2013; Murakami et al., 2014; Laprovitera et al., 2018). However, these methods generally require long analysis time, specialized equipment, and long operation time (Ye et al., 2019). Thus, alternative approaches are needed to address current technology’s limitations and enable the ultrasensitive detection of miRNAs (Song et al., 2016).

Recently, the lateral flow assay (LFA) has developed rapidly and has been widely employed in food safety, disease diagnosis, drug detection, and environmental monitoring (Hu et al., 2017; Hu et al., 2018; Daniell et al., 2019; Kim H. T et al., 2021). Compared with other traditional methods, LFA showed the characteristics of high specificity, low cost, and convenient operation (Hong et al., 2018; Mahmoudi et al., 2020). Nevertheless, it was reported to be limited by two major aspects: low sensitivity and inability to analyze quantitatively (Bahadir and Sezginturk, 2016). Thus, numerous methods have been developed to solve the problems such as fluorescence, surface plasmon resonance, and chemiluminescence (Cui et al., 2015; Fan et al., 2020; Kim K et al., 2021). However, these developed methods have not satisfied the detection limit and precision applicable to clinical practice so far. Surface-enhanced Raman spectroscopy (SERS), a common trace detection method, has been widely used in biology, medical, environmental protection, and food detection because of its advantages of high sensitivity, fast speed, and low signal interference (Liu et al., 2017; Sun et al., 2017; Neng et al., 2020; Nowicka et al., 2021). SERS enhancement effect was mainly attributed to the combination of electromagnetic mechanism (EM) and chemical mechanism (CM), which referred to the local surface plasmon resonance (LSPR) and electron transfer between adsorbed molecules and nanomaterials, respectively (Xu et al., 2018; Yu et al., 2020). Because EM was widely accepted to be the main reason for SERS signal enhancement, the selection of nanomaterials will become the key factor of SERS enhancement (Zhao et al., 2020). In recent years, palladium (Pd)–gold (Au) core–shell nanorods (Pd-AuNRs) has shown a wide range of application prospects that have been widely studied due to its unique and adjustable LSPR properties, excellent catalytic efficiencies, and SERS efficiencies. The presence of tips and edges on the surface of the rod-like nanostructure can serve as “hot spots” for large electric field enhancement, resulting in strong SERS signals. The core–shell setting borrows high SERS activity from the Au core through the long-range electromagnetic enhancement in addition to the enhancement from the Pd shell itself, which could lead to the transfer of plasma-induced hot electrons between the bimetallic interface, redistributing the local field around Pd and Au; thus, the electric field around Pd-AuNRs could significantly increase. Compared with AuNRs, Pd-AuNRs contain more intra- and interparticle gaps, tips, and edges, which are supposed to be rich in “hot spots,” giving the strong SERS signals at extremely low concentrations. Furthermore, the aggregation of nanoparticles can generate a more significant electromagnetic field coupling than that of individual nanoparticles.

SERS-LFA biosensors have been generally applied for the detection of hormone, nucleic acids, and proteins because it solved the problems such as low sensitivity and lack of precision in quantitative evaluation caused by conventional LFA biosensors (Hwang et al., 2016; Wang et al., 2017; Tran et al., 2019). However, SERS-LFA biosensors still cannot realize the ultrasensitive detection for some low concentration targets. It is pretty necessary to combine the SERS-LFA biosensor with a signal amplification strategy, such as hybridization chain reaction (HCR), rolling circle amplification (RCA), and catalytic hairpin assembly (CHA) (Li et al., 2010; Huang et al., 2021; Jain et al., 2021). Although all of these methods could greatly enhance the signal intensity, HCR suffered from laborious labeling techniques and was environmentally sensitive. Meanwhile, RCA showed a risk for sample contamination, which could add complexity and limit the efficiency. As an emerging DNA signal amplification technique, CHA could be performed at room temperature with low background signals when used to detect nucleic acid targets, which caused its universal application in the quantitative detection of small nucleic acid molecules and proteins. Hence, introducing CHA to SERS-LFA biosensors could significantly increase the signal intensity, realizing the ultrasensitive detection of miRNAs. However, dynamic monitoring of LSCC-related miRNAs in serum with a CHA-assisted SERS-LFA biosensor has not been reported, which plays pivotal roles in the early diagnosis, the prognostic assessment, and the selection and monitoring of proper treatment modalities.

In this study, a neoteric SERS-LFA biosensor combining CHA was developed to carry out the ultrasensitive and simultaneous detection of two LSCC-associated miRNAs (miR-106b and miR-196b) rapidly. The SERS tags (Pd-AuNRs@4-MBA@bio-HP_1-1_@MIgG and Pd-AuNRs@NBA@bio-HP_1-2_@MIgG) were synthesized by modifying the Raman molecules [4-mercaptobenzoic acid (4-MB) and nile blue A (NBA)] and hairpin DNA (bio-HP_1-1_ and bio-HP_1-2_) onto the Pd-AuNRs surface. During the detection process, miR-106b and miR-196b could be captured by SERS tags and a CHA reaction could be triggered. To better detect the target miRNAs, several key experiment conditions were optimized, including the volume of SERS tags for miR-106b, the volume of SERS tags for miR-196b, the concentration of the two hairpin DNA (HP_2-1_ and HP_2-2_), and the category of buffer solution and incubation time. Then, several interferences were introduced to evaluate the selectivity of the proposed SERS-LFA biosensor. The biosensors prepared at different times were used to assess the reproducibility, and then, the uniformity was verified by SERS mapping. The sensitivity was evaluated by testing the SERS intensities on the test lines under different concentrations of target miRNAs. Finally, this method was applied for to quantitatively detect target miRNAs in serum obtained from patients with LSCC at different stages to verify its feasibility. It has been confirmed that the SERS-LFA biosensor coupling with CHA amplification strategy provided a rapid and sensitive method for miRNAs detection, showing a great clinical application prospect for the early diagnosis of cancers.

## Materials and Methods

### Chemicals and Reagents

Sodium borohydride (NaBH_4_), hexadecyl trimethyl ammonium bromide (CTAB), chloroauric acid tetrahydrate (HAuCl_4_), sodium oleate (NaOL), ammonium sulfate [(NH_4_)_2_SO_4_], Pd chloride (PdCl_2_), hydrochloric acid (HCl), sodium hydroxide (NaOH) ascorbic acid, tris acetate (C_6_H_15_NO_5_), and silver nitrate (AgNO_3_) were purchased from Sinopharm Chemical Reagent Co. Ltd. (China). 4-MBA, NBA, carbodiimide (EDC), bovine serum albumin (BSA), tris-2-carboxyethyl phosphine (TCEP), N-hydroxysuccinimide (NHS), and Tris-HCl were obtained from Sigma-Aldrich. The quantitative RT-PCR (qRT-PCR) kit, streptavidin (SA), goat anti-mouse antibody (GMIgG), and mouse monoclonal antibody (MIgG) were provided by Getein Biotech (China). The ultrapure water used for all experiment was prepared on a Milli-Q (Millipore, USA, >18 M) purifier. All the beakers and magnetic stir bars were cleaned in the new-made aqua regia (HCl/HNO_3_, 3:1) and then rinsed with ultrapure water thoroughly followed by drying. All reagents were from commercial sources and used without further purification. Gelose, 6×DNA loading buffer, 50×TAE buffer, GelRed dye solution, and DL15000 DNA Marker were purchased from Linc-Bio Science Inc. (China). As presented in Table 1, the oligonucleotides were provided by Minneapolis (USA).

**TABLE 1:** Sequences of oligonucleotides used in the experiment.

Name	Sequences (5′-3′)
bio-HP_1-1_	HS-ATCTGCACTGTCAGCACTTTACGACATCTAACTAAAGTGCTGACAG-biotin
bio-HP_1-2_	HS-CCCAACAACAGGAAACTACCTACGACATCTAACTAGGTAGTTTCCTG-biotin
HP_2-1_	GCA​CTT​TAG​TTA​GAT​GTC​GTA​AAG​TGC​TG ACAGCGA CATCTAAC
HP_2-2_	ACT​ACC​TAG​TTA​GAT​GTC​GTA​GGT​AGT​TTC​CTG​CGA​CAT​CTA​AC
MT1-1	UAA​AGU​GCU​GAG​AGU​GCA​GAU
MT1-2	UAG​GUA​GUU​UCC​UGC​UGU​UGG​G
MT3-1	UAA​ACU​GGU​GAC​AGU​GCC​GAU
MT3-2	UAC​GUA​GUU​UGC​UCU​UGU​UGG​G
Random	ACG​GCC​UUA​CGU​ACG​AAC​CCG
miR-106b	UAA​AGU​GCU​GAC​AGU​GCA​GAU
miR-196b	UAG​GUA​GUU​UCC​UGU​UGU​UGG​G

### Collection and Processing of Serum Samples

A total of 150 serum specimens were collected from 30 healthy subjects and 120 patients with LSCC from the College of Clinical Medicine of Yangzhou University. Ⅰ, Ⅱ, Ⅲ, and Ⅳ represent different stages of LSCC, and there were 30 patients at each stage. All serum specimens were obtained on the day of diagnosis and were collected in EP tubes after centrifugation (300 rpm, 10 min) at 4°C, labeling corresponding to the classification followed by −80°C storage. Detailed information was summarized in Table 2. The whole experiments were performed in accordance with the guidelines of “Declaration of Helsinki” and approved by the ethics committee at the Yangzhou University. Informed consent was obtained from the human participants of this study.

**TABLE 2:** Characteristics of the participants in the study.

Groups	Healthy subjects	Ⅰ	Ⅱ	Ⅲ	Ⅳ
Gender					
Male	15	14	13	18	15
Female	15	16	17	12	15
Average age	27	32	39	46	54
Sample	30	30	30	30	30

### Synthesis of Pd-AuNRs

Au nanorods (AuNRs) were synthesized *via* the reported seed growth method using the binary surface active mixture of CTAB and NaOL (Ye et al., 2013). The synthesis steps were as follows: first, 250 μl of HAuCl_4_ (10 mM) was added to 9.75 ml of CTAB (0.1 M). After stirring for 15 min, 0.8 ml of fresh ice was added to bath-prepared NaBH_4_ (10 mM) rapidly followed by stirring vigorously for 2 min. Then, the seed solution was stored at 28°C for 30 min. Subsequently, 3.6 g of CTAB and 0.494 g of NaOL were dissolved in 200 ml of ultrapure water followed by adding 10 ml of HAuCl_4_ (10 mM) under the magnetic agitation. When the solution became colorless, 4 ml of AgNO_3_ (10 mM) was added and 0.6 ml of HCl was injected after stirring for 5 min and 320 μl of AA (0.1 M) was then mixed with the above solution. After about 30 s, 0.5 ml of the prepared seed solution was added with constant stirring and then stored the mixture at 40°C for 12 h. Then, the mixture was centrifuged (9,000 rpm, 20 min) and cleaned twice, and the resulting AuNRs was dispersed in 10 ml of ultrapure water. Subsequently, 180 μl was mixed with 1 ml of CTAB (0.1 M) followed by centrifugation after 1 h ultrasound. Precipitation was added with 300 µl of CTAB (0.1 M) and then 50 μl of the resulting solution was mixed with 1 ml of CTAB (20 mM). After sitting for 10 min, 10 μl of CuCl_2_ (2 mM) and 25 μl of H_2_PdCl_4_ (10 mM) were added, and 12.5 μl of AA (0.1 M) was added 5 min later followed by even shaking and then stored at 30°C for 12 h. Finally, the mixture was centrifuged (9,000 rpm, 10 min) twice and dispersed in pure water; thus, Pd-AuNRs could be obtained.

### Synthesis of Two SERS Tags

Briefly, 100 μl of 4-MBA (1 mM) solution was added to 2 ml of Pd-AuNRs solution, respectively, and reacted at room temperature for 1 h under magnetic agitation (650 rpm). Then, TCEP was used to activate the hairpin DNA (bio-HP_1-1_): 30 μl of bio-HP_1-1_ (0.1 M) was mixed with 40 μl of the newly prepared TECP (1 mM) buffer for 1 h followed by mixing the activated bio-HP_1-1_ with 1 ml of 4-MBA–labeled Pd-AuNRs for 12 h. Subsequently, 40 μl of EDC (160 mM) and 40 μl of NHS (35 mM) were added to the mixture and then 200 μl of MIgG (8 μg/μl) was added after incubating for 2 h. Following that, the resulting solution was dispersed in 20 μl of BSA solution (1 wt%) and incubated for 60 min. Finally, the SERS tag (Pd-AuNRs@4-MBA@bio-HP_1-1_@MIgG) could be obtained after purifying the mixture by centrifugation (9,000 rpm, 10 min) and resuspending in the PBS buffer. Replacing the signal molecule and hairpin DNA to NBA and bio-HP_1-2_, and the other SERS tag (Pd-AuNRs@NBA@bio-HP_1-2_@MIgG), could be synthesized after repeating the above procedure.

### Preparation of SERS-LFA Strip

The lateral flow strip was consist of four parts: the sample pad, the conjugate pad, the nitrocellulose (NC) membrane (two test lines and one control line), and the absorption pad. To increase its surface activity and hydrophilicity, the sample pad was pretreated with a buffer solution containing 100 mM Tris-HCl, 0.5% TRITON®X-100, and 300 mM NaCl followed by drying at 37°C for 2 h. Then, the conjugate pad was treated with a buffer solution consisting of Tween 20 (0.2 wt%), BSA (5 wt%), and sucrose (2 wt%). After drying at room temperature, the synthesized SERS tags was pipetted onto the as-prepared conjugate pad and dried at 37°C for 3 h. During the preparation process of NC membrane, 4 μl of HP_2-1_ (1 μM) and 15 μl of SA (0.4 mg/ml) were sprayed onto the test line one (T1) at a rate of 0.6 μl/cm. Then, 5 μl of HP_2-2_ (10 μM) and 15 μl of SA (0.4 mg/ml) were sprayed onto the test line two (T2) at the same rate. The control line (C line) was prepared by spaying 5 μl of GMIgG (1:50 dilution) onto the NC membrane. A plastic backing pad was used to assemble the lateral flow strip and, at all the four parts, a 2-mm overlap was existed to make sure that the sample solution could flow through the lateral flow strip. Finally, the integrated strips were cut into 4-mm-wide strips and stored in a sealed bag at 4°C in a dry state.

### SERS Measurement

During the test process, 100 μl of the sample solution was dropped onto the sample pad at about 5 mm from the end. Then, the sample solution could flow toward the direction of the absorbent pad along with the SERS tags (Pd-AuNRs@4-MBA@bio-HP_1-1_@MIgG and Pd-AuNRs@NBA@bio-HP_1-2_@MIgG) by capillary action. In addition, the whole LFA process could be completed within 25 min, and a visible gray band could appear on the C line. The SERS spectra on T1 (or T2) line were recorded at 785-nm excitation wavelength with a 50× long working distance objective. The laser power and acquisition time were set as 5 mW and 10 s, respectively. To eliminate the noises of the instrument, all the SERS spectrum was treated with baseline correction and smoothing. To achieve the quantitative analysis of target miRNAs and ensure the validity and representativeness of the results, SERS spectrum was the average result gained from 15 different spots on the test lines.

### Instrumentation

The UV-visible absorption spectra were recorded with an Agilent Cary UV-5000 UV-vis spectrophotometer (USA). Transmission electron microscopy (TEM) images were characterized by Tecnai 12 TEM (Philips, Netherlands). Scanning electron microscopy (SEM) images were obtained with an S-4800II field emission SEM (Hitachi, Japan). High-resolution TEM (HRTEM) images, high-angle annular dark-field scanning TEM (HAADF-STEM) images, and selected area electron diffraction (SAED) were captured by Tecnai G2 F30 s-twin TEM (FEI, USA). Raman spectra of test lines were obtained on an inVia micro-Raman spectrometer (Renishaw, UK).

## Results and Discussion

### Design of Detection Scheme for SERS-LFA Biosensors

**FIGURE 1:**
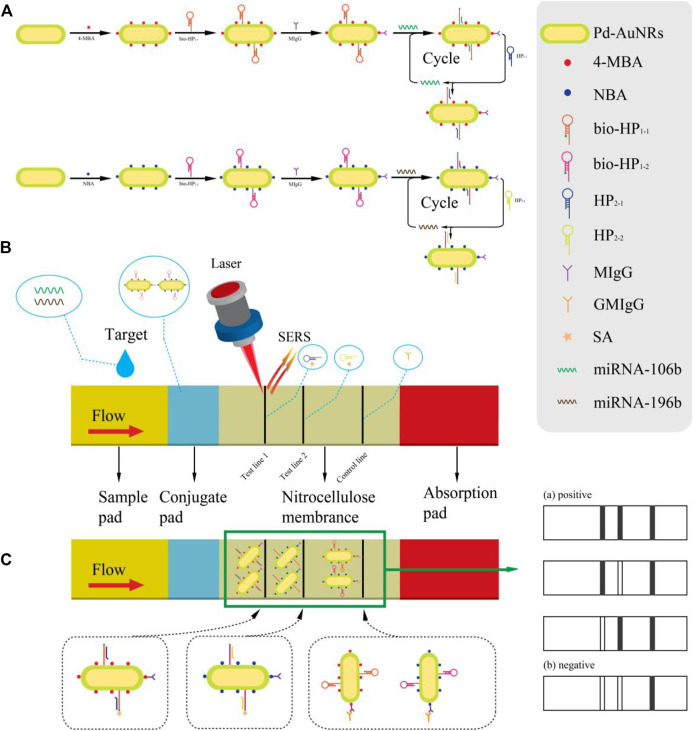
**(A)** Schematic diagram of the synthesis procedure of two SERS tags and the principle of corresponding CHA reaction. **(B)** A clear illustration for the assembly of the test strips and the SERS-LFA biosensor for detecting miR-106b and miR-196b simultaneously. **(C)** The principle of double-positive result and diagrams of other possible result.

The working principle of the synchronous detection of miR-106b and miR-196b with the proposed SERS-LFA biosensor is presented in Figure 1. Figure 1A is the diagram of the preparation process of SERS tags and the corresponding CHA reaction principle. *Via* the Au–S bond, 4-MBA and NBA were modified to the surface of Pd-AuNRs as the Raman reporters. Because of the presence of sulfhydryl groups at the 5′ end of hairpin DNA (bio-HP_1-1_ and bio-HP_1-2_), it could be connected to the surface of Pd-AuNRs with the Au–S bond. The carboxyl groups of 4-MBA and NBA could be activated by coupling agents (EDC and NHS); thus, the monoclonal antibodies (MIgG) could be conjugated to 4-MBA and NBA under the action of a stable amide bond. At this point, the two SERS tags (Pd-AuNRs@4-MBA@bio-HP_1-1_@MIgG and Pd-AuNRs@NBA@bio-HP_1-2_@MIgG) were prepared, which were complementary to the target miRNAs (miR-106b and miR-196b), respectively. *Via* a toehold-mediated strand displacement reaction, bio-HP_1-1_ (or bio-HP_1-2_) on the corresponding SERS tag was opened by miR-106b (or miR-196b) followed by hybridizing it, and biotin could be exposed toward the outside. The next stage of the CHA reaction was triggered by HP_2-1_ (or HP_2-2_), and the bio-HP_1-1_@HP_2-1_ (or bio-HP_1-2_@HP_2-2_) composite structure could be generated *via* another toehold-mediated strand displacement reaction.

Figure 1B presents the detection process, and serum collected from healthy subjects and patients with LSCC at different stages were dripped onto the sample pad, migrating through the capillary action. When passing the conjugate pad, the CHA reaction was triggered due to the presence of target miRNAs (miR-106b or miR-196b). As the solution flowed to the NC membrane along the strip, the next stage of the CHA reaction was activated by HP_2-1_ (or HP_2-2_) fixed on the T1 (or T2) line. Thus, the bio-HP_1-1_@HP_2-1_ (or bio-HP_1-2_@HP_2-2_) composite structure could be formed with biotin molecules exposed outside which were captured by SA on the T1 (or T2) line *via* the specific binding between SA and biotin (Xia et al., 2021). As the reaction went on, the SERS tags were fixed on the test lines constantly, significantly amplifying the signal intensity, and then, two strong gray lines could be presented on the test line. Subsequently, excessive complex continued to flow through the C line, and the complex could be captured by the prefixed anti-human IgG (GMIgG) *via* the principle of antigen-antibody binding (Gu et al., 2018). Then, the whole detection process was finished, and three strong gray lines were formed on the strip (Figure 1C). Figure 1C also shows the four possible results of the SERS-LFA biosensor, including three positive and one negative result. If miR-106b and miR-196b existed at the same time, then three visible gray lines would displayed on the strip. If only miR-106b or miR-196b existed, then the two gray lines would be displayed. If no target miRNAs existed, then only C line would show a distinct gray line. The distinguishable characteristic peaks of 4-MBA and NBA could be recorded by the Renishaw Raman microscope, realizing the quantitative analysis of miR-106b and miR-196b.

### Characterization of Pd-AuNRs

**FIGURE 2:**
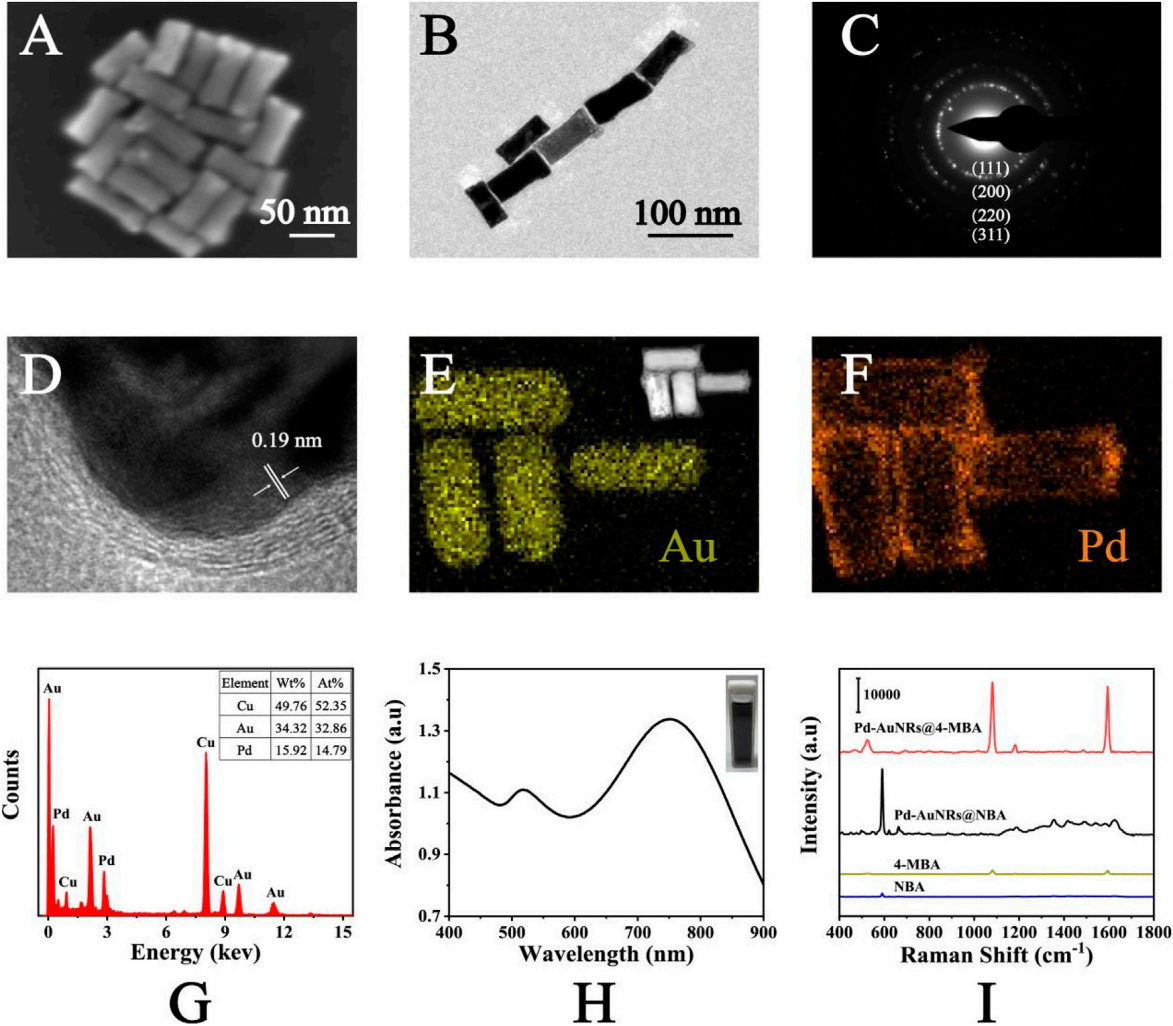
**(A)** SEM images and **(B)** TEM images of Pd-AuNRs. **(C)** SAED pattern and **(D)** HRTEM images of Pd-AuNRs. **(E)** HAADF-STEM image and corresponding EDS elemental mapping of the Au and **(F)** Pd elements of the Pd-AuNRs. **(G)** The EDS spectrum of the Pd-AuNRs. **(H)** UV-Vis-NIR absorption spectrum of Pd-AuNRs. **(I)** SERS spectra of NBA-labeled Pd-AuNRs, 4-MBA–labeled Pd-AuNRs, pure NBA solution, and pure 4-MBA solution.

SEM and TEM images were applied for the characterization of Pd-AuNRs nanostructure. Figure 2A shows the representative SEM images of the Pd-AuNRs, indicating its satisfactory dispersion and uniform morphology. TEM images (Figure 2B) of Pd-AuNRs demonstrated that the average particle length was 70 nm and the average particle width was 30 nm. To study the crystalline structure of Pd-AuNRs, HRTEM images were obtained. The fringes in a typical HRTEM image (Figure 2D) were separated by 0.19 nm, in great agreement with the {100} lattice spacing of Pd. The side facets of AuNR seeds were composed of four {110} facets and four {100} facets, having a cylindrical shape. During the growth of the Pd shell, the competition growth between the {110} and {100} side facets lead to the disappearance of {110} facets, resulting in a Pd shell with four {100} side facets. SAED pattern was taken to further investigate the morphology of Pd-AuNRs (Figure 2C). It clearly revealed that Pd-AuNRs grew randomly in orientations such as {111}, {200}, {220}, and {311}. The energy-dispersive X-ray spectroscopy (EDS) mapping in Figures 2E,F indicated that Pd-AuNRs were composed of dense AuNRs core and sparse Pd shell, corresponding to the HAADF-STEM in the illustration of Figure 2E. The EDS spectrum in Figure 2G further demonstrated that Pd-AuNRs were composed of Au elements and minor Pd elements, which accounted for 34.32% (wt%) and 15.92% (wt%), respectively. The appearance of Cu elements was due to the copper net used to load Pd-AuNRs for HRTEM and EDS analysis. Figure 2H presents the UV-Vis-NIR absorption spectrum of Pd-AuNRs, having two distinct absorption peaks at 748 and 519 nm.

As shown in Figure 2I, SERS spectra of NBA (1 × 10^−2^ M), 4-MBA (1 × 10^−2^ M), NBA-labeled Pd-AuNRs (1 × 10^−6^ M), and 4-MBA–labeled Pd-AuNRs (1 × 10^−6^ M) was recorded. The characteristic peak of NBA was observed at 592 cm^−1^, caused by the in-plane vibration of CCC and CNC of NBA (Alvarez-Puebla et al., 2009). The characteristic peaks observed at 1,080 and 1,594 cm^−1^ were ascribed to the in-plane ring breathing coupled with v (C-S) and v (C-C) modes of 4-MBA (Talley et al., 2004). The SERS spectra showed that the signal intensities of NBA-labeled Pd-AuNRs and 4-MBA–labeled Pd-AuNRs were much strong than that of two Raman reporters. Thus, it demonstrated that Pd-AuNRs had a significant enhancement effect, and its analytical enhancement factor (AEF) was calculated referring to the following equation: AEF = (I_SERS_/C_SERS_)/(I_RS_/C_RS_), where I and C represent the normalized Raman intensity and the concentration of analyte, respectively. The calculated AEF for Pd-AuNRs was 6.31 × 10^6^. This satisfactory SERS performance mainly attributed to the gaps, tips, and edges contained in Pd-AuNRs and the redistribution of the local electric field around Pd and Au caused by the core–shell spatial structure.

### Characterization of SERS Tags

**FIGURE 3:**
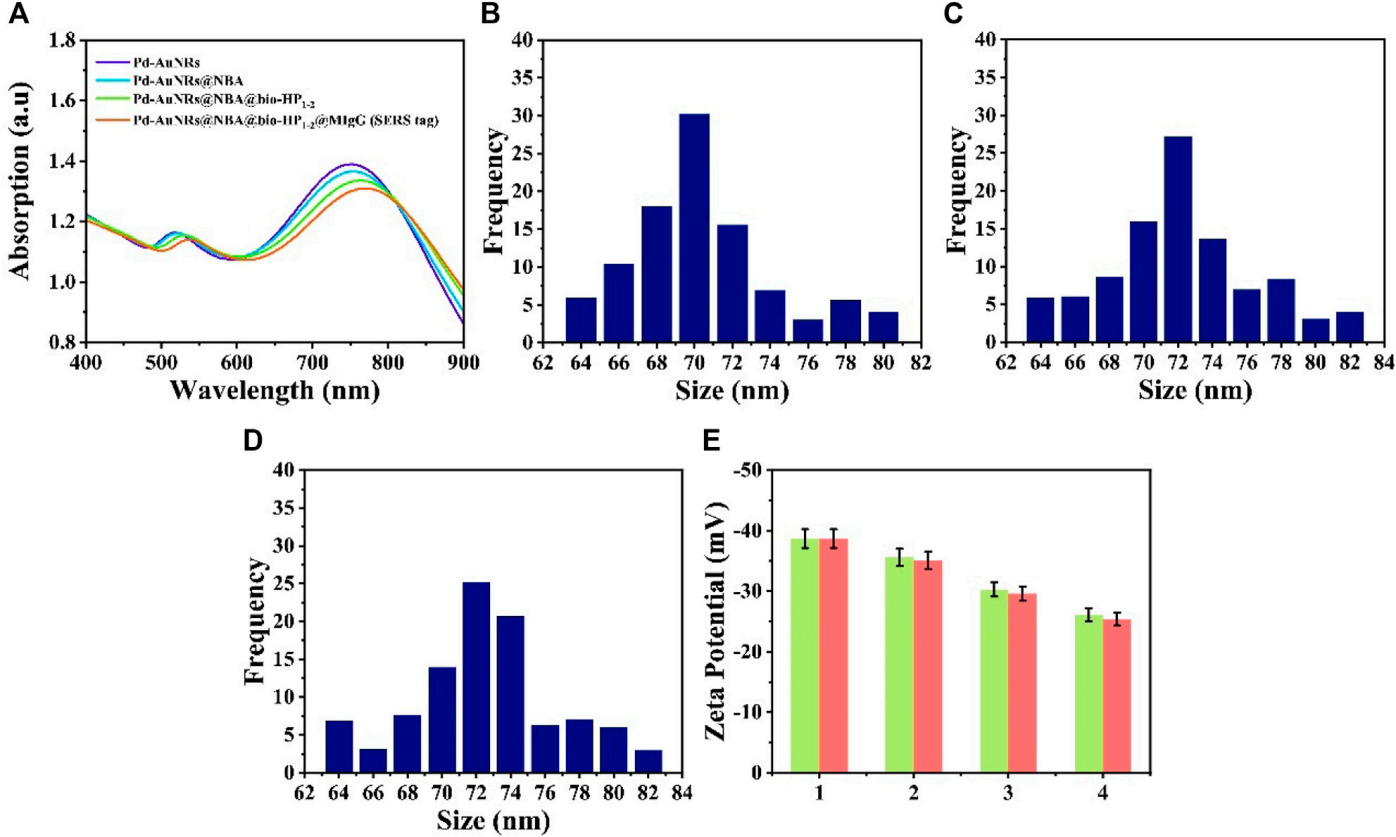
**(A)** UV-Vis-NIR spectra of Pd-AuNRs, Pd-AuNRs@NBA, Pd-AuNRs@ NBA@bio-HP_1-2_, and Pd-AuNRs@NBA@bio-HP_1-2_@MIgG (SERS tag). Size distribution histogram of **(B)** Pd-AuNRs and the composite structures after modifying **(C)** 4-MBA and **(D)** NBA. **(E)** Zeta potential analysis: 1 represents Pd-AuNRs and 2, 3, and 4 represents the composite structures after modifying with 4-MBA (NBA), bio-HP_1-1_ (bio-HP_1-1_), and MIgG.

SERS tag (Pd-AuNRs@NBA@bio-HP_1-2_@MIgG) was composed of four functional structure: Pd-AuNRs acting as the enhancement substrate, NBA acting as the reporter molecule, bio-HP_1-2_ serving as one of the CHA reactants for enhancing the SERS signal, and MIgG serving as the anchor point for GMIgG fixed on the C line. Figure 3A shows the UV-Vis-NIR absorption spectra of synthetic process for Pd-AuNRs@NBA@bio-HP_1-2_@MIgG. The spectra of the pure Pd-AuNRs colloids presented two strong LSPR bands at about 748 and 519 nm due to the plasmon resonance. After the assembly of NBA molecules, bio-HP_1-2_, and MIgG on the surface of Pd-AuNRs, a decrease could be caused and plasmon resonance at 748 nm was red-shifted to 755, 764, and 775 nm, respectively. The decrease and red-shift of the SPR peak may be caused by the slight aggregation of Pd-AuNRs during the assemble process. The characterization of Pd-AuNRs@4-MBA@bio-HP_1-1_@MIgG was likewise. To better understand the formation process of SERS tags, the changes in particles size and zeta potential upon addition of 4-MBA and NBA. As displayed in Figures 3B–D, the addition of 4-MBA and NBA increased the size of the Pd-AuNRs by approximately 2 nm. Figure 3E (green charts) shows that, with the addition of 4-MBA, the zeta potentials increased from −38.7 to −35.6 mV, and then, they further increased to −30.3 mV after the addition of bio-HP_1-1_ and were maintained at approximately −26.1 mV upon the addition of MIgG and BSA. The red charts represented the zeta potentials of synthesis process of Pd-AuNRs@NBA @bio-HP_1-2_@MIgG. Although the zeta potentials for the SERS tags were slightly higher than −30 mV, which was considered to represent the boundary of stable suspension, they were observed to be quite stable.

### FDTD Simulation of Pd-AuNRs

**FIGURE 4:**
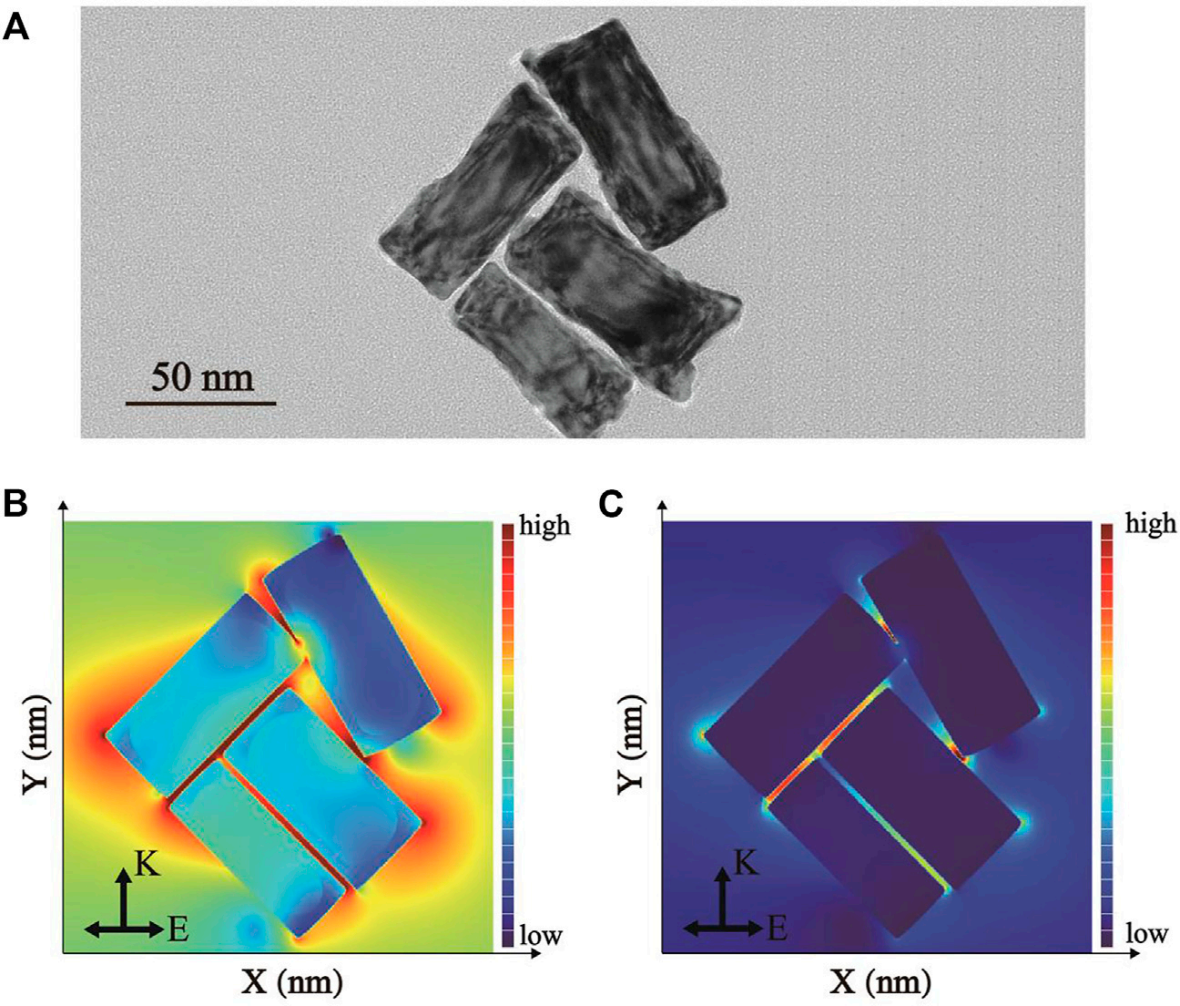
**(A)** TEM images of Pd-AuNRs tetramer. The simulated electric field intensity distribution images of Pd-AuNRs when **(B)** E_Z_ = 0 nm and **(C)** E_y_ = 0 nm.

The finite-difference time-domain (FDTD) simulation was applied as an effective approach to further study the spatial electric field distribution of synthesized Pd-AuNRs under the irradiation of a beam of linearly polarized light. The complex refractive indexes of Au and Pd were adopted from the refractive index database in the simulation software packages [Au - CRC and Pd - Palik, respectively]. All geometric parameters for simulations were consistent with the average actual size of as-prepared samples shown in TEM image. To maintain the accuracy and effectiveness of the FDTD simulation, the model was constructed in the form of TEM images (Figure 4A) with four Pd-AuNRs. Herein, boundary conditions were set as perfectly matched layers in all simulations. Figures 4B,C present the spatial electric field intensity distribution images of Pd-AuNRs when E_Z_ = 0 nm and E_y_ = 0 nm, respectively. The field intensity enhancement widely exists around the Pd-AuNRs, and the largest enhancement was located at the tips and edges, which significantly more than that of AuNRs. In this way, the enhancement of Pd-AuNRs was much larger than that of corresponding unshelled AuNR. Moreover, the electromagnetic field enhancements located between adjacent Pd-AuNRs were more significant than those around single Pd-AuNR. Therefore, the Pd-AuNRs aggregations during the LFA process could generate more “hot spots,” which could greatly enhance the signal intensity.

### Qualitative Analysis of miR-106b and miR-196b

**FIGURE 5:**
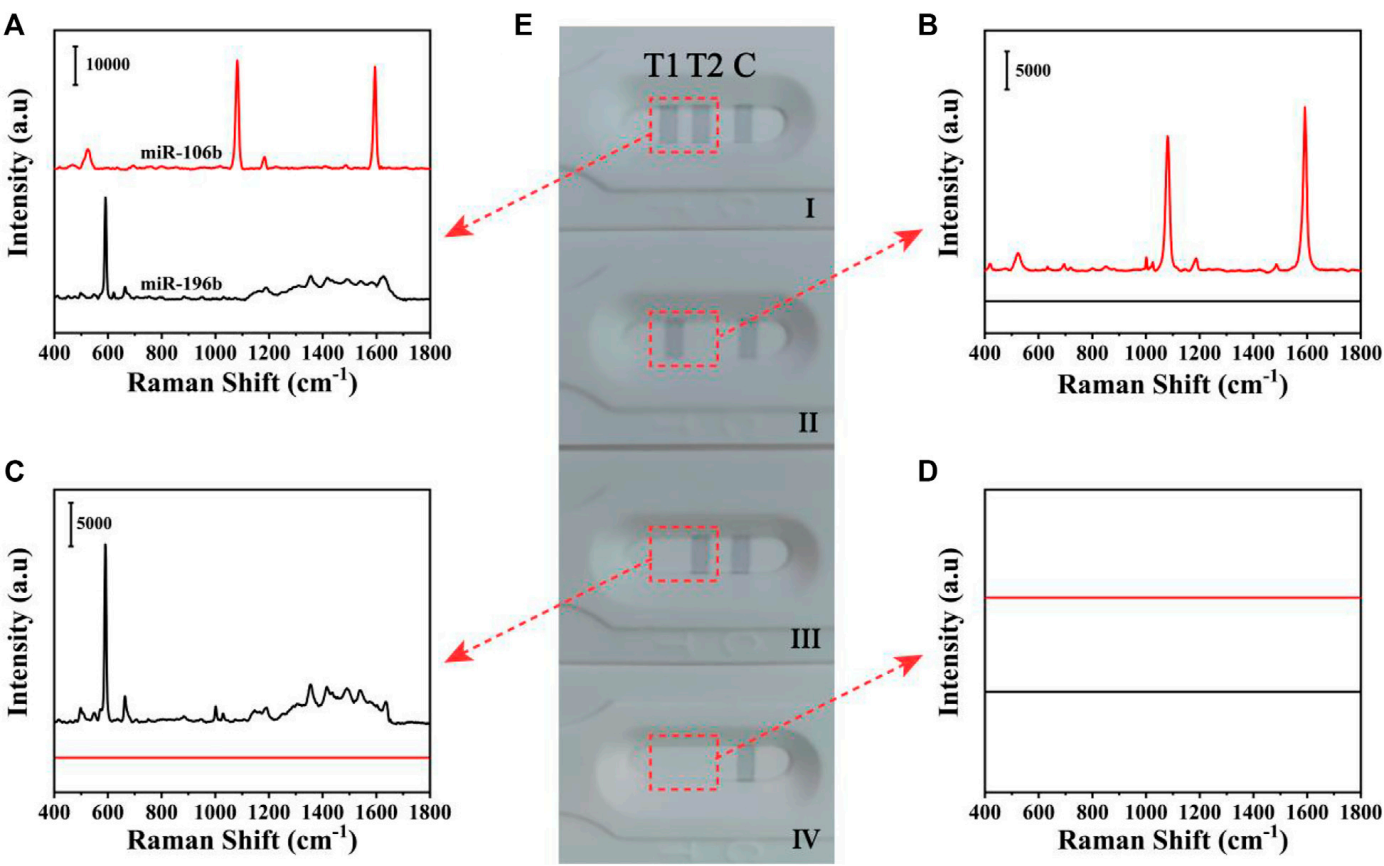
The SERS spectra of four different results of the detection of miR-106b and miR-196b on the T1 line and T2 line **(A–D)**. **(E)** The corresponding digital photo of four different results.

The qualitative analysis of miR-106b and miR-196b simultaneously on the basis of the SERS-LFA biosensor was presented, and obvious color changes could be observed on the test lines. Figure 5 shows the photographs of different detection results and corresponding SERS spectra on the test lines. Because the GMIgG absorbed on the C line could capture the SERS tags *via* the antigen-antibody binding principle, a strong gray line could consistently appear regardless of the presence of miR-106b and miR-196b; thus, the C line on the LFA strips could be used to verify the accuracy of the results. In the presence of miR-106b and miR-196b with a concentration of 1 nM, three distinct gray lines appeared on the strip (Figure 5Ei) and two visible characteristic peaks at 1,080 and 592 cm^−1^ from 4-MBA and NBA could be distinguished in the corresponding SERS spectra (Figure 5A). When only miR-106b or miR-196b included in the sample, only two gray lines appeared on the strip (Figures 5Eii,iii) and only one SERS spectra was presented (Figures 5B,C). When no target miRNAs existed, only one gray line appeared on the strip (Figure 5Eiv), and no SERS signal could be recorded (Figure 5D). For four different results, the SERS spectra changes were consistently with the color changes, proving that the SERS-LFA biosensor could be applied for the qualitative analysis of miR-106b and miR-196b.

### Cross-Reactivity Analysis

**FIGURE 6:**
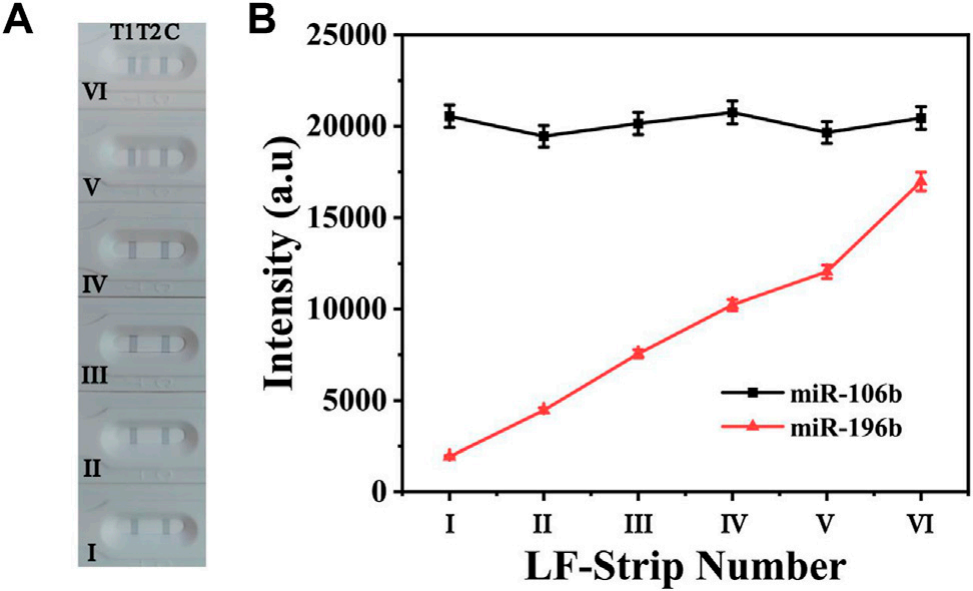
**(A)** The digital photo and **(B)** corresponding SERS intensities of the two test lines at 1,080 and 592 cm^−1^. The concentration of the applied sample solution: (i) 100 aM miR-196b + 100 pM miR-106b; (ii) 1 fM miR-196b + 100 pM miR-106b; (iii) 10 fM miR-196b + 100 pM miR-106b; (iv) 100 fM miR-196b + 100 pM miR-106b; (v) 1 pM miR-196b + 100 pM miR-106b; (vi) 10 pM miR-196b + 100 pM miR-106b.

To evaluate the cross-reactivity between two target miRNAs, 100 pM miR-106b was mixed with miR-196b at different concentrations ranging from 100 aM to 10 pM. The digital photo of the detection results is shown in Figure 6A; it clearly demonstrated that T1 line showed the same degree of gray color for 100 pM miR-106b in all samples, proving that the color intensity change for T2 line could not affected the T1 line. In contrast, a light gray color was presented on T2 line (Figure 6Avi) for 10 pM miR-196b, and no obvious color changes could be observed when the concentration was lower than 1 pM. The corresponding line chart (Figure 6B) showed that the intensities at 592 cm^−1^ increased with the miR-196b ranging from 100 aM to 10 pM, and only little change could be found on the intensities of 4-MBA at 1,080 cm^−1^, with the deviation calculated as 6.72%. Furthermore, another two interferences were introduced (Supplementary Figure S1) and the deviation was calculated as 5.03% and 5.69%, respectively. Thus, the experimental results could not be influenced by cross-reactivity.

### Feasibility Evaluation of CHA-Assisted SER-LFA Biosensor

**FIGURE 7:**
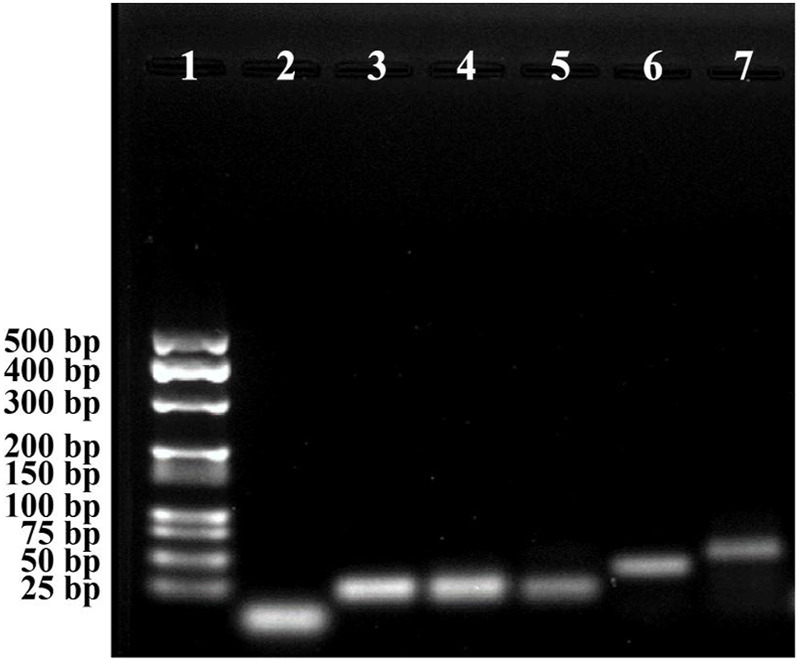
Gel electrophoresis of the CHA-assisted SER-LFA biosensor for the detection of miR-106b. Lane 1, Marker; Lane 2, HP_1-2_; Lane 3, HP_2-2_; Lane 4, target miRNAs (miR-106b); Lane 5, HP_1-2_ + HP_2-2_; Lane 6, HP_1-2_+target miRNA; Lane 7, HP_1-2_ + HP_2-2_ + target miRNA.

The feasibility of the CHA-assisted SER-LFA biosensor was evaluated by the gel electrophoresis. As displayed in Figure 7, the exhibited bands in lane 5 (HP_1-2_+HP_2-2_) was almost a simple superposition of lane 2 and lane 3, and no significant hybridization products could be observed at the top of lane 5, indicating that HP_1-2_ could not react with HP_2-2_ when no target miRNA existed. When incubated target miRNA with HP_1-2_, the band of HP_1-2_ disappeared and a new band of large molecular weight complex appeared, demonstrating that target miRNA could trigger the open of hairpin. After mixing HP_2-2_ with HP_1-2_+target miRNA, a strong band of HP_1-2_-HP_2-2_ complex appeared and the target miRNA was released, confirming the successful occurrence of CHA. This result indicated that the CHA-assisted SER-LFA biosensor could be applied to detect some low concentration targets because CHA could enhance the signal significantly.

### Optimization of SERS-LFA Biosensor Parameters

**FIGURE 8:**
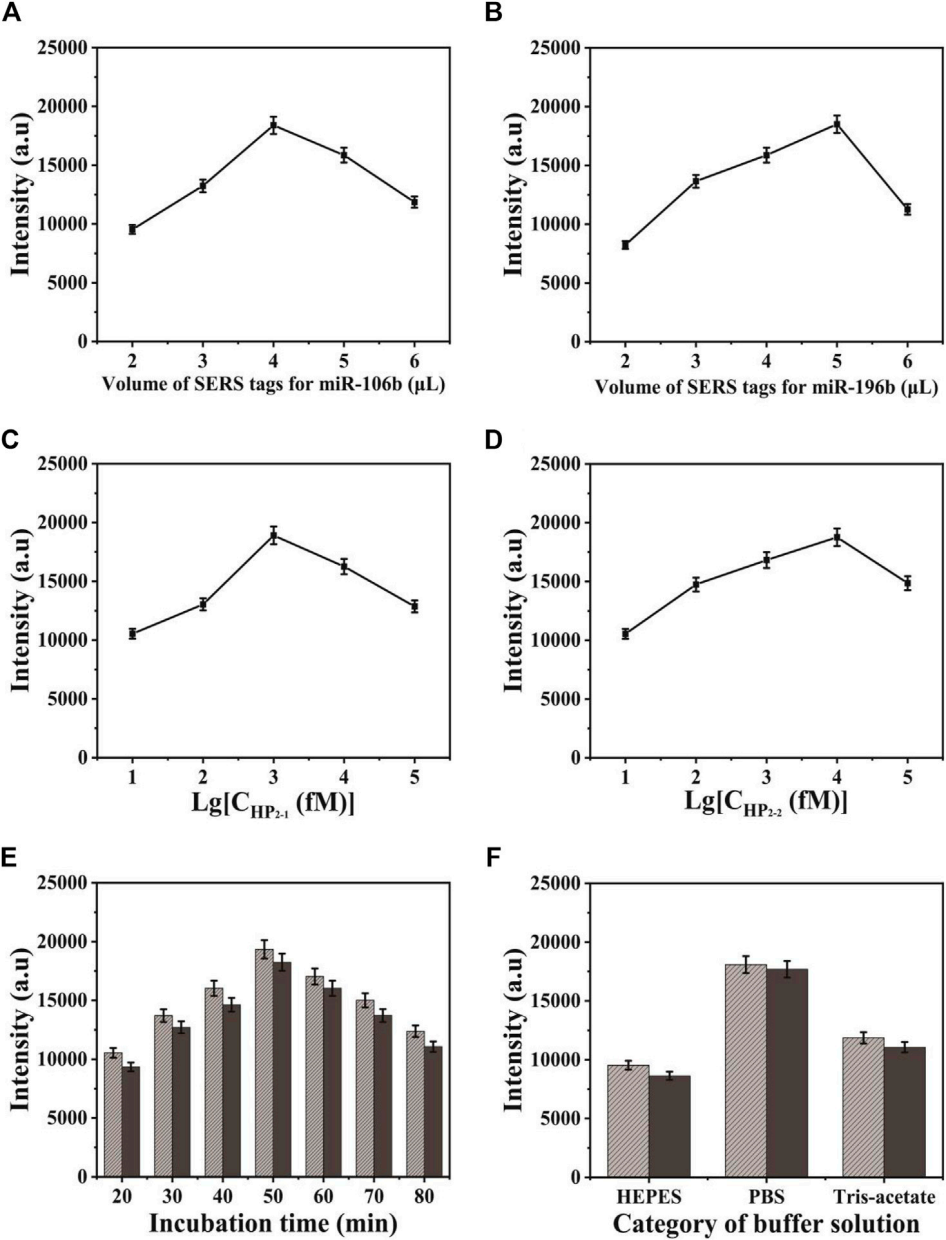
Optimization of different detection conditions on SERS intensity: **(A)** the volume of SERS tags for miR-106b; **(B)** the volume of SERS tags for miR-196b; **(C)** the concentration of HP_2-1_; **(D)** the concentration of HP_2-2_; **(E)** the incubation time of the strip after adding the target miRNAs; and **(F)** the category of buffer solution.

In the process of SERS-LFA biosensor fabrication, several conditions were investigated, including the volume of SERS tags for miR-106b and miR-196b, the concentration of HP_2-1_ and HP_2-2_, and the category of buffer solution and incubation time, to obtain the optimal experimental conditions. Herein, the characteristic peaks of NBA and 4-MBA at 592 and 1,080 cm^−1^ were employed to quantify the SERS intensities. The volume of two SERS tags for target miRNAs were investigated in Figures 8A,B because it could affect the background signal. It could be indicated that the SERS intensities increased gradually along with the volume increase of SERS tags for miR-106b when the amount was lower than 4 μl. As the amount of SERS tags for miR-106b gradually increased, SERS intensities decreased gradually because it contributed more to the background signal, compared to SERS signal. Thus, the optimal amount of SERS tags for miR-106b was chosen to be 4 μl. Similarly, the amount of SERS tags for miR-196b was optimized to 5 μl. Because CHA could enhance the SERS signal significantly, it is quite necessary to optimize the CHA parameters. As presented in Figures 8C,D, the concentration of HP_2-1_ and HP_2-2_ was optimized. When the concentration of HP_2-1_ was lower than 1 pΜ, the SERS intensities increased gradually. When the concentration of HP_2-1_ was more than 10 pΜ, the intensities decreased due to the significantly enhanced background signal; thus, the optimal concentration of HP_2-1_ was 1 pΜ. In the same way, the concentration of HP_2-2_ was optimized to 10 pΜ. As presented in Figure 8E, the SERS intensities increased gradually from 20 to 50 min and then decreased slightly from 50 to 80 min. Thus, 50 min was selected for the optimal incubation time in the following experiment. To obtain the best analytical performance of SERS-LFA biosensor, several buffer solutions were investigated including HEPES, PBS, and Tris-Acetate. As shown in Figure 8F, PBS presented the highest SERS intensity. Thus, PBS was applied as the experimental buffer solution.

### Evaluation of Selectivity, Reproducibility, and Uniformity

**FIGURE 9:**
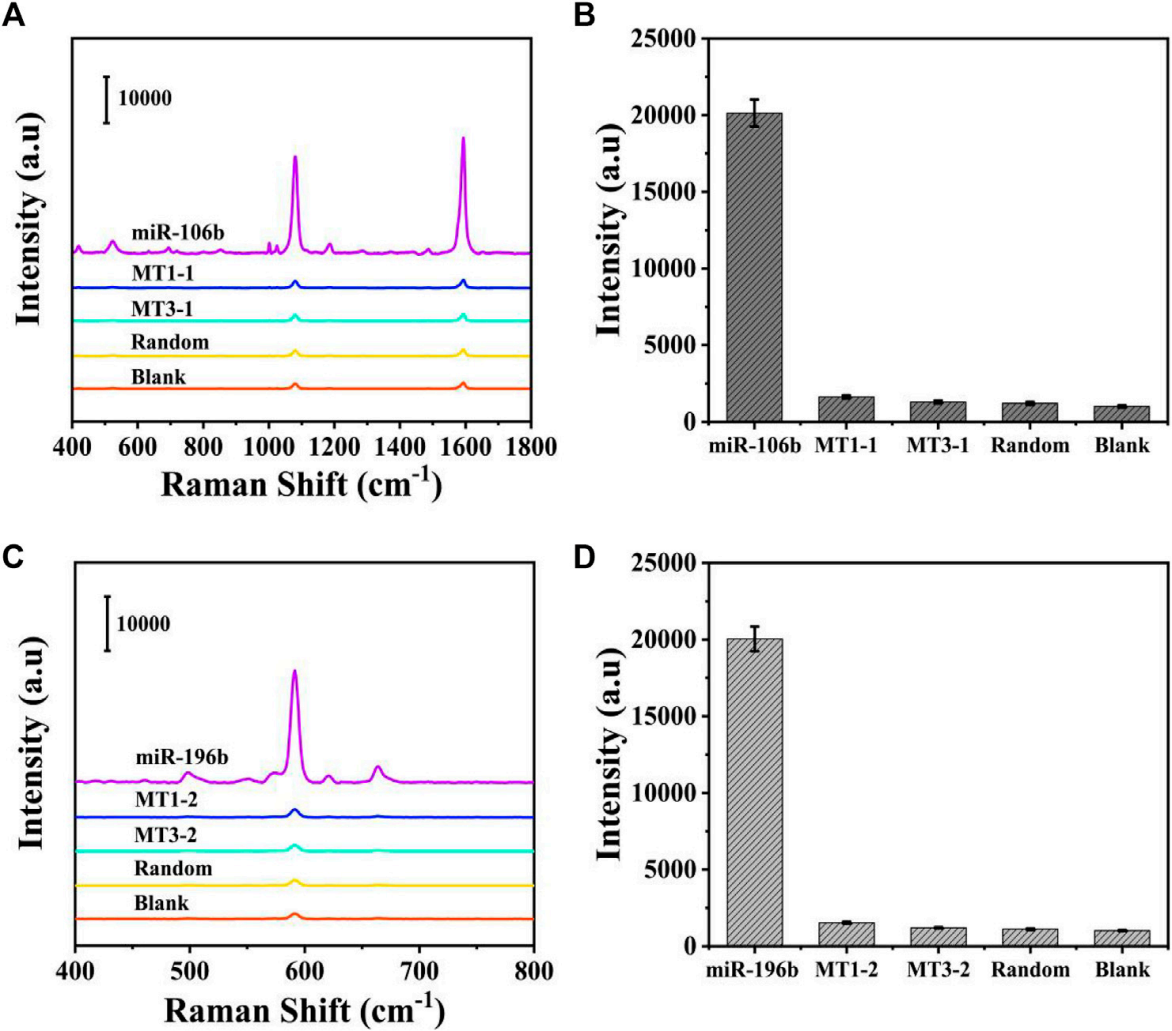
Selectivity of the SERS-LFA biosensor. **(A)** SERS spectra of the analytes at the same concentration (100 pM): miR-106b, MT1-1, MT3-1, random, and blank. **(B)** The corresponding bar chart of SERS intensities at 1,080 cm^−1^. **(C)** SERS spectra of the analytes at the same concentration (100 pM): miR-196b, MT1-2, MT3-2, random, and blank. **(D)** The corresponding bar chart of SERS intensities at 592 cm^−1^.

Because the selectivity was quite important for the accurate detection, it was investigated by introducing several interferences, including one-base mismatch (MT1) sequence, three-base mismatch (MT3) sequence, and random sequence under the optimized conditions. The concentration of miR-106b, miR-196b, and interferences was set to 100 pM in PBS buffer. As illustrated in Figures 9A,C, the SERS intensities of miR-106b and miR-196b were more significant than that of the interferences and blank control. Then, the characteristic peaks at 1,080 and 592 cm^−1^ were employed to quantify the SERS intensities in Figures 9B,D, respectively. Because the low peak intensities of the interferences and blank control could be ignored and the target miRNAs could be distinguished effectively, the SERS-LFA biosensor was proved to show a satisfied selectivity, showing a great application prospect in the mixed samples.

**FIGURE 10:**
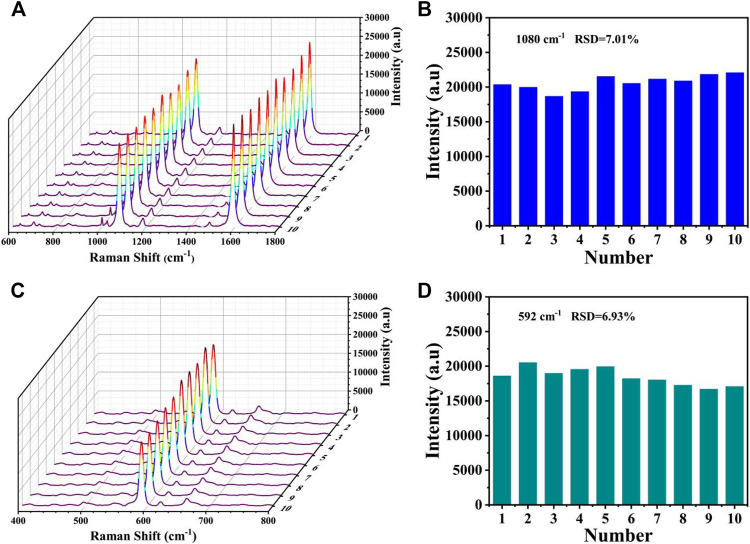
Reproducibility of the SERS-LFA biosensor. **(A)** SERS spectra of 10 SERS-LFA biosensors prepared at different batches for the detection of miR-106b and **(B)** corresponding bar chart of the SERS intensities at 1,080 cm^−1^. **(C)** SERS spectra of 10 SERS-LFA biosensors prepared at different batches for the detection of miR-196b and **(D)** corresponding bar chart of the SERS intensities at 592 cm^−1^.

The reproducibility was also evaluated by measuring the SERS spectra from the T1 lines (4-MBA) and T2 lines (NBA) of the biosensors prepared at 10 different baths under the same experiment condition. As displayed in Figures 10A,C, only slight differences in intensities could be observed in the SERS spectra, and the shapes almost existed no obvious difference. The corresponding bar charts of the SERS intensities at 1,080 and 592 cm^−1^ was presented in Figures 10B,D, respectively. The relative standard deviations (RSDs) were calculated as 7.01% and 6.93%, respectively, further proving its prominent reproducibility.

**FIGURE 11:**
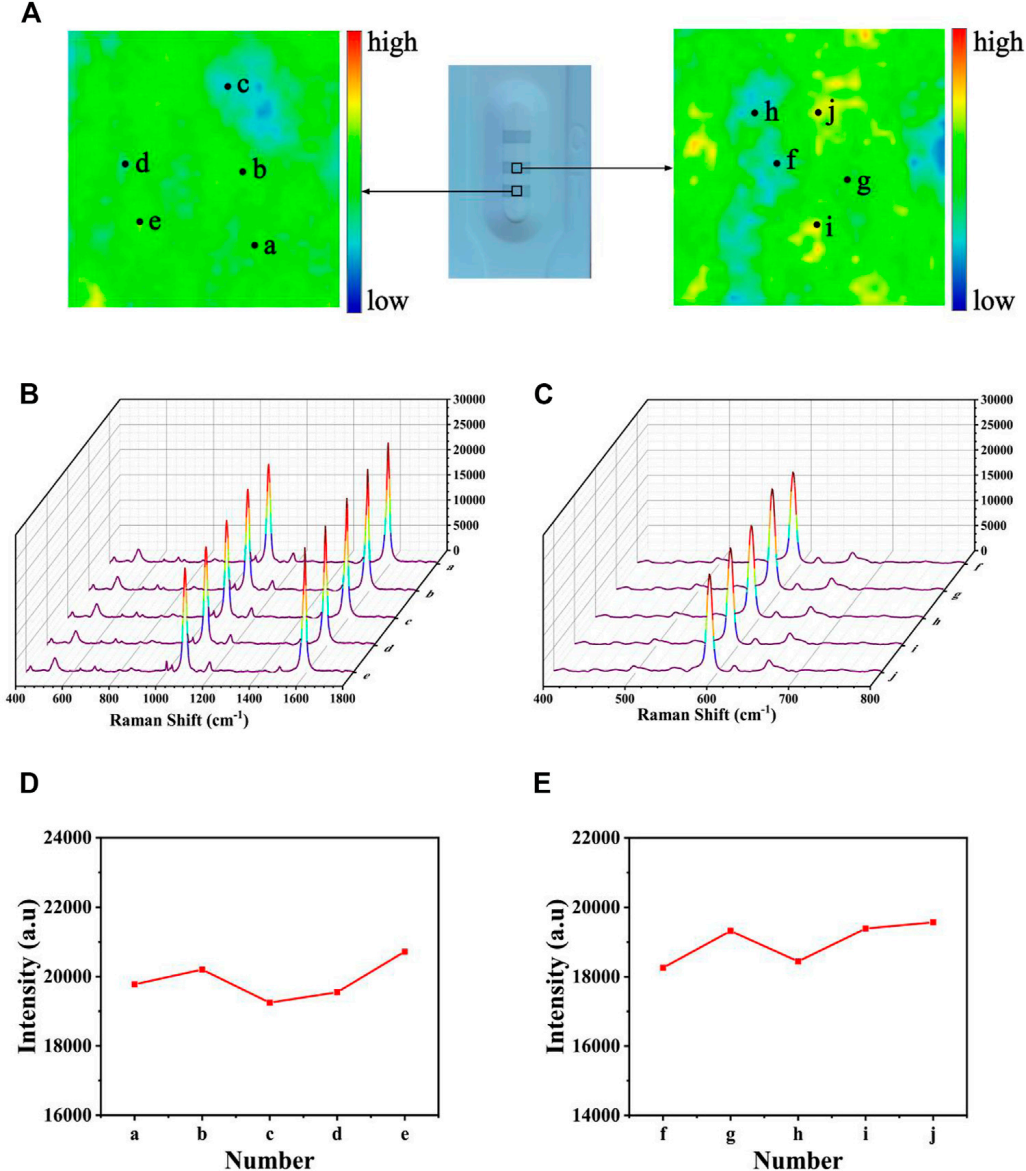
Uniformity of the SERS-LFA biosensor. **(A)** SERS mapping of 4-MBA and NBA on the SERS-LFA strip. **(B)** SERS spectra of five randomly selected point regions on the T1 line and **(D)** corresponding line chart of intensities at 1,080 cm^−1^. **(C)** SERS spectra of five randomly selected point regions on the T2 line and **(E)** corresponding line chart of intensities at 592 cm^−1^. Stability of the SERS-LFA biosensor.

Uniformity is one of the most important factors affecting the precision of the detection; thus, it was quite important to assess the uniformity of test lines. After modifying 4-MBA and NBA, respectively, a SERS mapping was performed on the T1 (and T2) line (Figure 11A), with a scanning range of 50 × 50 mm. The scheme of color ranging from red (maximum intensity) to blue (minimum intensity) was employed. Each grid point represented the SERS intensities at 1,080 and 592 cm^−1^. Although some areas still existed as blue or red, most of them were uniformly green, proving the great uniformity of the proposed SERS-LFA biosensor. Subsequently, 10-point regions were selected randomly on the test lines, and the SERS spectra was recorded in Figures 11B,C. Obviously, no significant difference could be observed between the spectra, only a slight difference in the intensities. Figures 11D,E presented the corresponding line chart of the SERS intensities at 1,080 and 592 cm^−1^ respectively, and the deviation of signal intensities was calculated as 6.19% and 8.36%, respectively. To sum up, the proposed SERS-LFA biosensor showed great selectivity, reproducibility, and uniformity, which were beneficial to the detection of miR-106b and miR-196b.

**FIGURE 12:**
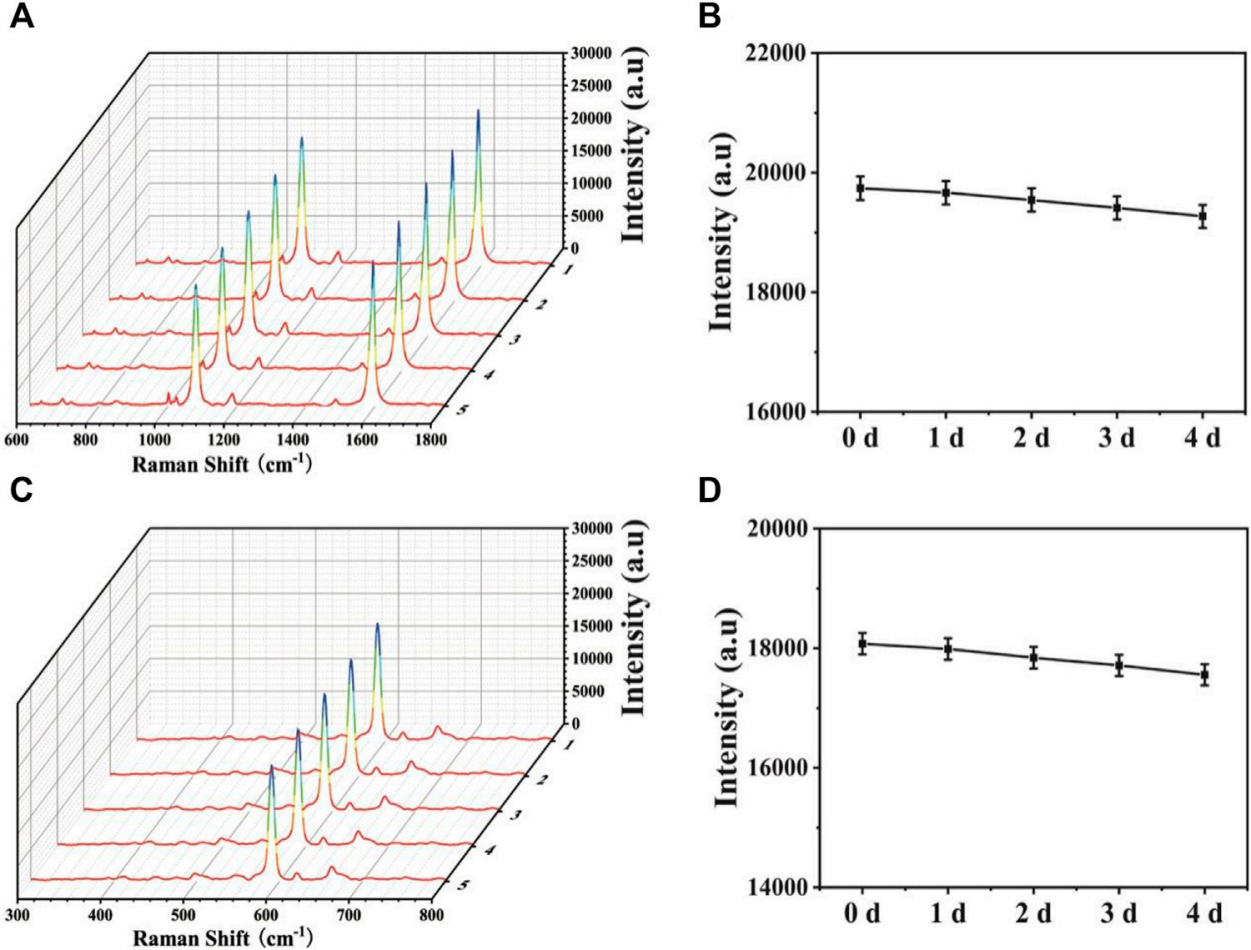
SERS spectra of **(A)** miR-106b and **(B)** miR-196b in PBS buffer with different concentrations (100 aM, 1 fM, 10 fM, 100 fM, 1 pM, 10 pM, 100 pM, and 1 nM) and corresponding calibration curves of SERS intensities at **(C)** 1,080 and **(D)** 592 cm^−1^.

To ensure the wide application, stability is another important point to assess the SERS-LFA biosensors. Thus, SERS spectra of the proposed SERS-LFA biosensor after the 1-, 2-, 3-, and 4-day storage were recorded. As displayed in Figures 12A,C, only slight intensity decline could be observed and the shapes almost demonstrated no obvious difference. Figures 11B,D presented intensities at 1,080 and 592 cm^−1^, respectively, and the deviations were calculated as 5.19% and 6.25%, respectively. Therefore, the proposed SERS-LFA biosensor presented satisfactory stability.

### Quantitative Detection of Target miRNAs Simultaneously

The sensitivity for the quantitative and simultaneous detection of miR-106b and miR-196b was assessed under the optimized conditions. MiR-106b and miR-196b were dissolved together in PBS buffer and diluted to different concentrations ranging from 100 aM to 1 nM. Figure 12 shows the photograph of the biosensor in the presence of various target miRNAs concentrations, and it was difficult to identify colorimetric changes of test lines for miR-106b and miR-196b at concentrations lower than 100 fM and 1 pM, respectively. As the target miRNAs concentrations increased, more sandwich complexes are formed, and the gray color became more and more visible. SERS spectra of the test lines was recorded in Figures 12A,C, respectively. It clearly demonstrated that the SERS intensities increased with the increasing concentrations of miR-106b and miR-196b. Moreover, the corresponding calibration curves were displayed in Figures 12B,D, where y represents the SERS intensities at 1,080 and 592 cm^−1^, respectively, and x represents the logarithm of the miR-106b and miR-196b concentrations, respectively. The equation obtained from linear regression in Figure 11B is y = 2810.91 × −4230.79 with a correlation coefficient (*R* ^2^) of 0.99025. Similarly, the equation in Figure 11D is y = 2823.55 × −3767.08, with *R* ^2^ = 0.99377. Thus, the limits of detection (LODs) for miR-106b and miR-196b in PBS could be calculated as 23.17 and 46.94 aM, respectively.

**FIGURE 13:**
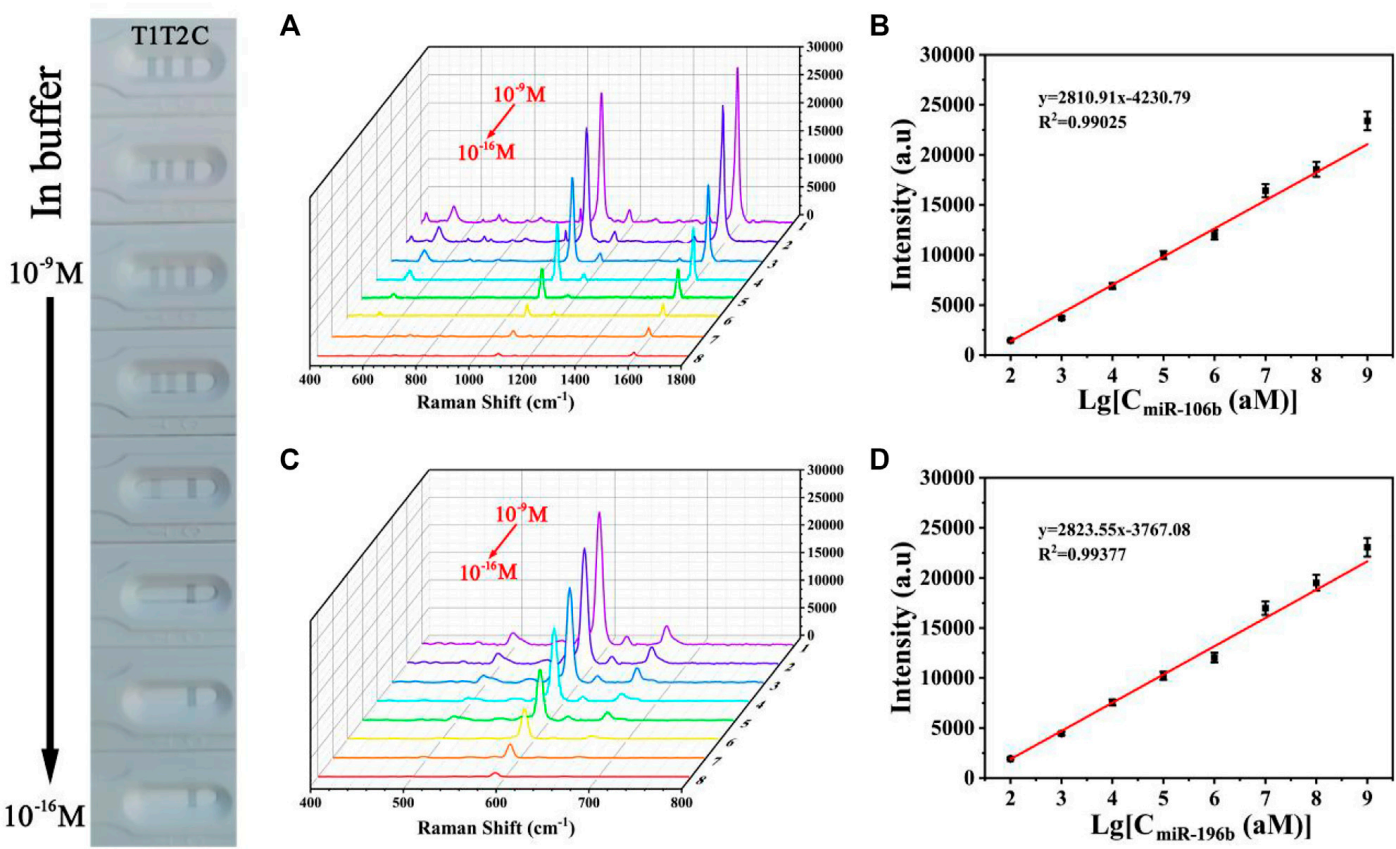
SERS spectra of **(A)** miR-106b and **(B)** miR-196b in human serum with different concentrations (100 aM, 1 fM, 10 fM, 100 fM, 1 pM, 10 pM, 100 pM, and 1 nM) and corresponding calibration curves of SERS intensities at **(C)** 1,080 and **(D)** 592 cm^−1^.

To assess the sensitivity of the developed SERS-LFA biosensor in human serum, miR-106b and miR-196b were mixed with human serum and diluted to different concentrations (100 aM to 1 nM) followed by detecting by the SERS-LFA biosensor. As the increasing concentration of miR-106b and miR-196b, SERS intensities increased gradually, and the gray color of the test lines on the strips (Figures 13A,C) became obvious gradually. A great linear relationship was found between the logarithm of miR-106b concentration and the SERS intensity at 1,080 cm^−1^. The equation obtained from linear regression in Figure 13B is y = 2679.61 × −3937.63 with a correlation coefficient (*R* ^2^) of 0.98906. Similarly, Figure 13D shows that the linear relationship was found between the logarithm of miR-196b concentration and the SERS intensity at 592 cm^−1^, and the equation is y = 2764.35 × −3862.81, with *R* ^2^ = 0.99386. Moreover, the LOD for miR-106b and miR-196b in human serum was calculated as 43.08 and 61.36 aM, respectively. As illustrated in Table 3, the sensitivity in this work was compared to that in other works, and it clearly demonstrated that this proposed method had lower LOD than most reported methods. Therefore, the developed SERS-LFA biosensor possessed excellent sensitivity for the detection of miRNAs in PBS and human serum, showing great application prospects in clinical diagnosis.

**TABLE 3:** Comparison of the proposed SERS-LFA biosensor to other methods.

Method	Analyte	LOD (M)	Ref.
Fluorescence	miR-155	3.34 × 10^−14^	Miao et al. (2018)
Colorimetry	miR-29a-3p	1.0 × 10^−12^	Miao et al. (2016)
Colorimetry	miR-155	6.0 × 10^−10^	Borghei et al. (2018)
Electrochemistry	miR-21	8.43 × 10^−14^	Rafiee-Pour et al. (2016)
SERS	miR-132-3p	1.0 × 10^−14^	McArdle et al. (2017)
SERS	miR-106b	4.308 × 10^−17^	This work
	miR-196b	6.136 × 10^−17^	

### Accuracy Verification

**FIGURE 14:**
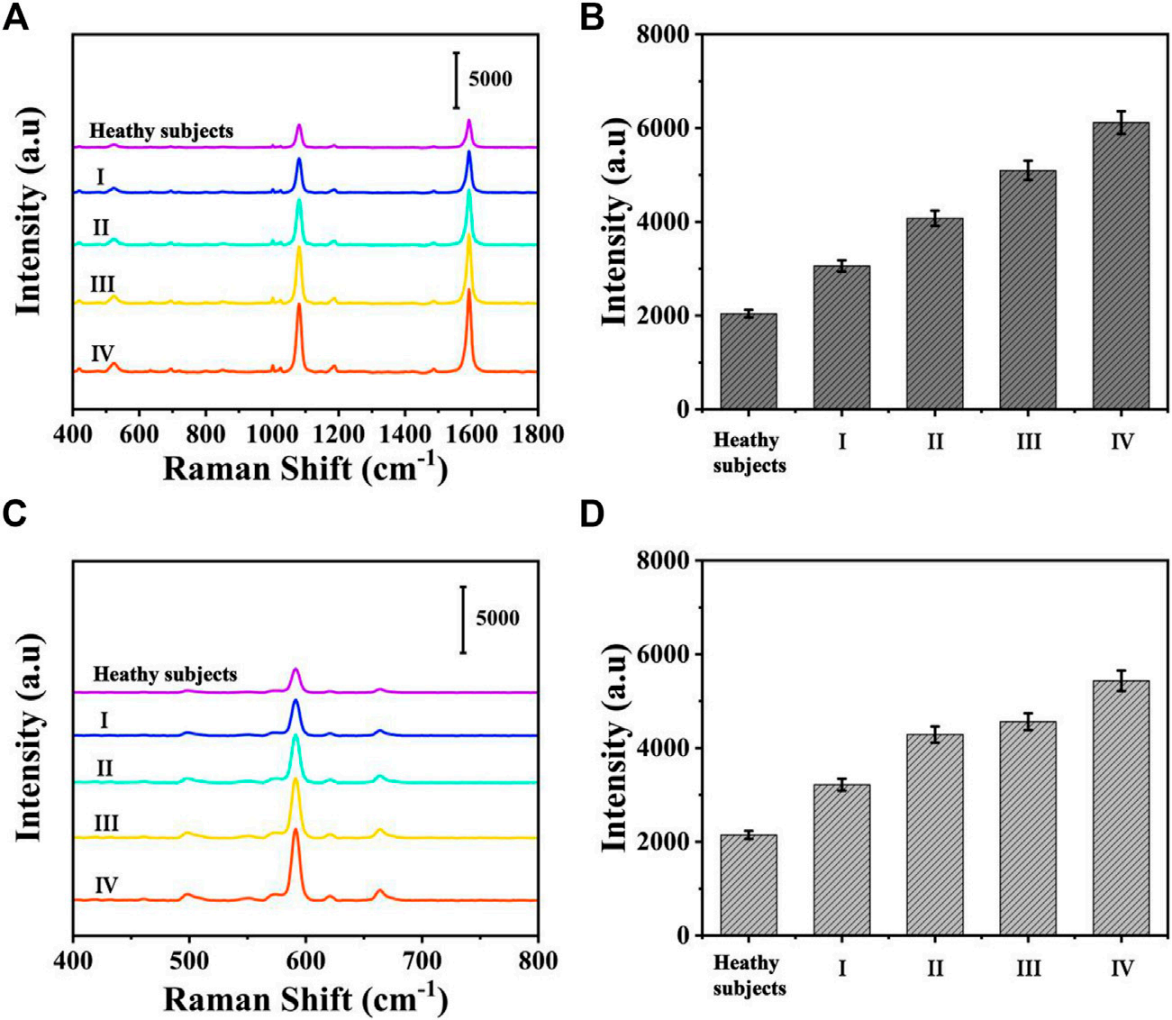
The average SERS spectra of **(A)** miR-106b and **(C)** miR-196b in human serum at different stages and corresponding bar chart of SERS intensities at **(C)** 1,080 and **(D)** 592 cm^−1^.

To assess the feasibility of the proposed SERS-LFA biosensor for real sample determination, SERS was applied to detect miR-106b and miR-196b levels in serum obtained from healthy subjects and patients with LSCC at different stages, and qRT-PCR was introduced to verify the accuracy. The test results of the two methods and the RSD of each group were shown in Supplementary Figures S2, S3 and Supplementary Tables S1–S5 (supporting information). Figures 14A,C present the average SERS spectra of miR-106b and miR-196b that corresponded to test lines, and each spectrum was the average of the actual spectra. The corresponding bar charts of SERS intensities were showed in Figures 14B,D, respectively. It clearly indicated that SERS intensities increased along with the cancer development, which was consistent with the miRNA’s levels. Then, the SERS intensities were substituted into the equation presented in Figures 13B,D to calculate the concentration of miR-106b and miR-196b (Table 4). The concentrations of miR-106b measured by the SERS-LFA biosensor were 0.181, 0.389, 0.921, 2.181 and 5.096 fM, whereas those measured by qRT-PCR were 0.174, 0.373, 0.881, 2.065, and 4.896 fM. Meanwhile, the relative errors were calculated as 3.867%, 4.113%, 4.343%, 5.318%, and 3.925%, respectively. The concentrations of miR-196b measured by the SERS-LFA biosensor were 0.144, 0.329, 1.066, 1.395, and 2.655 fM, whereas those measured by qRT-PCR were 0.139, 0.315, 1.011, 1.330, and 2.551 fM. The relative errors were calculated as 3.472%, 4.255%, 5.159%, 4.659%, and 3.917%, respectively. Obviously, no significant difference could be observed between the results obtained by the two methods, confirming the accuracy of the proposed SERS-LFA biosensor.

**TABLE 4:** Result of the SERS-LFA biosensor and qRT-PCR for the real samples.

Sample	SERS (fM)		qRT-PCR (fM)		Relative error (%)	
	miR-106b	miR-196b	miR-106b	miR-196b	miR-106b	miR-196b
Healthy subjects	0.181	0.144	0.174	0.139	3.867	3.472
Ⅰ	0.389	0.329	0.373	0.315	4.113	4.255
Ⅱ	0.921	1.066	0.881	1.011	4.343	5.159
Ⅲ	2.181	1.395	2.065	1.330	5.318	4.659
Ⅳ	5.096	2.655	4.896	2.551	3.925	3.917

## Conclusion

To sum up, Raman reporter–labeled Pd-AuNRs were employed to well establish the SERS-LFA biosensor assisted with CHA, allowing the ultrasensitive, selective, and reliable determination of LSCC-related miRNAs. The results demonstrated that the detection limit of miR-106b and miR-196b was low to 23.17 and 46.94 aM in PBS, and it could also be applied for the quantification of the target in serum collected from patients with LSCC, and the results were in excellent consistence with conventional qRT-PCR. The excellent performance of this method was ascribed to the following advantages: 1) Pd-AuNRs were synthesized on a large scale by a simple seed-mediated growth, with AuNRs as seeds. Pd-AuNRs contained abundant gaps, tips, and edges, serving as “hot spots” for large electric field enhancement, and the core–shell structure could redistribute the local electric field, resulting in strong SERS signals. 2) With the aid of CHA, Pd-AuNRs could aggregate on the T line and numerous nanometer gaps formed, further imparting a strong electromagnetic field enhancement. In addition, only target miRNAs can trigger the amplification reaction, realizing the high selective detection. 3) LFA exhibited desirable uniformity, stability, and reproducibility, which were necessary factors for the practical SERS application. 4) The whole detection process can be finished less than 1 h, enabling the dynamic monitoring of miRNAs in serum. These advantages give this approach a high potential for miRNAs quantification in clinical analysis.

## Data Availability

The original contributions presented in the study are included in the article/Supplementary Material, further inquiries can be directed to the corresponding authors.
