# Supplementary material for: SERS Based Lateral Flow Assay for Rapid and Ultrasensitive Quantification of Dual Laryngeal Squamous Cell Carcinoma-Related miRNA Biomarkers in Human Serum Using Pd-Au Core-Shell Nanorods and Catalytic Hairpin Assembly
Source: Front Mol Biosci. 2022 Feb 11;8:813007. doi: 10.3389/fmolb.2021.813007 (PMC8878268; doi:10.3389/fmolb.2021.813007)
Supplement: Supplementary file 1 [file DataSheet1.docx]

**Supporting Information**

# SERS based lateral flow assay for rapid and ultrasensitive quantification of dual laryngeal squamous cell carcinoma-related miRNA biomarkers in human serum using Pd-Au core-shell nanorods and catalytic hairpin assembly

Guang Li^a^, Ping Niu^c^, Shengjie Ge^d^, Dawei Cao*^b^, Aidong Sun*^a^

^a^Department of Otorhinolaryngology‑Head and Neck Surgery, The Affiliated Hospital of Yangzhou University, Yangzhou, 225001, P. R. China.

^b^College of Mathematics and Computer Science, Zhejiang Normal University, Jinhua 321004, P. R. China.

^c^Departments of Otolaryngology, The Affiliated Hospital of Shandong First Medical University, Qingzhou People’s Hospital, Qingzhou, 262500, P. R. China.

^d^Institute of Translational Medicine, Medical College, Yangzhou University, Yangzhou, 225001, P. R. China.

*Correspondence: [entsunaidong@163.com,](mailto:entsunaidong@163.com,) dwcsdfx@163.com

**1. Results and discussion**

**1.1 Cross-reactivity Analysis**


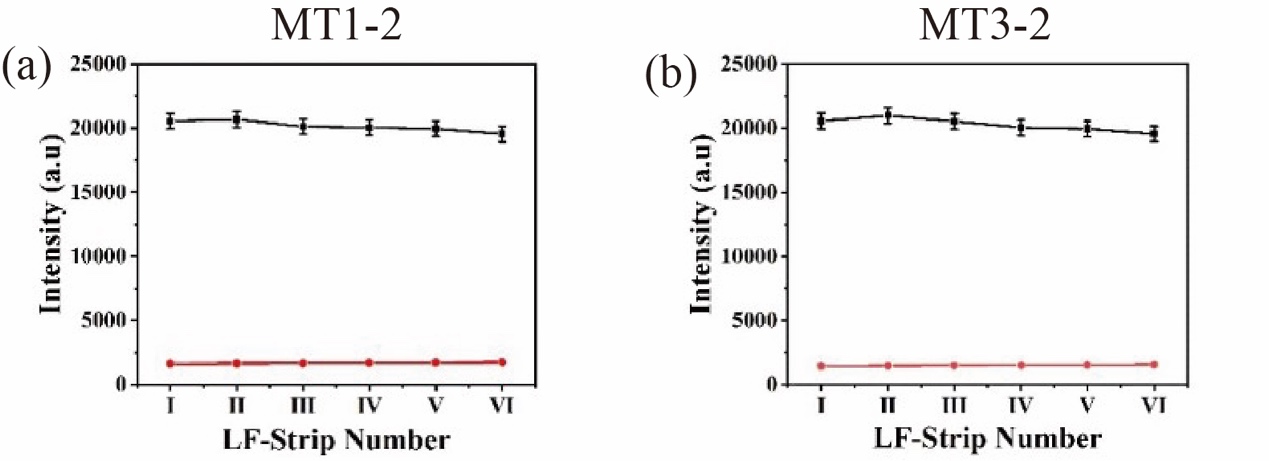


**FIGURE S1** SERS intensities of the two test lines at (a) 1080 cm^-1^ and (b) 592 cm^-1^. The concentration of the applied sample solution: (Ⅰ) 100 aM MT1-2 (MT3-2)+100 pM miR-106b; (Ⅱ) 1 fM MT1-2 (MT3-2)+100 pM miR-106b; (Ⅲ) 10 fM MT1-2 (MT3-2)+100 pM miR-106b; (Ⅳ) 100 fM MT1-2 (MT3-2)+100 pM miR-106b; (Ⅴ) 1 pM MT1-2 (MT3-2)+100 pM miR-106b; (Ⅵ) 10 pM MT1-2 (MT3-2)+100 pM miR-106b.

**1.2 Application analysis in real sample**

SERS spectra of miR-106b and miR-196b in serum obtained from healthy subjects and LSCC patients at different stages was presented in Fig. S2 and qRT-PCR was applied to verify the accuracy of test results. The difference in experimental results was mainly due to the randomness of sample selection. Table S1-S5 showed the results measured by the two methods. These results showed that the proposed SERS-LFA biosensor had high accuracy when used to detect practical samples.

**
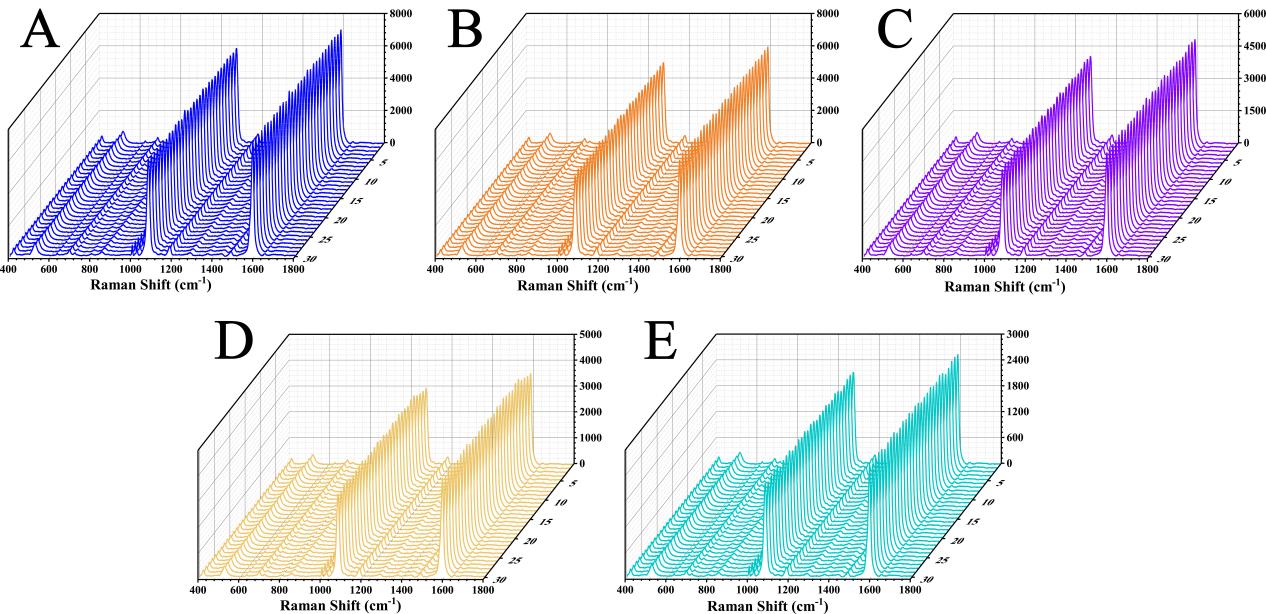
**

**Figure S2** SERS spectra of miR-106b in serum obtained from (A) healthy subjects, (B) LSCC patients at stage (B) Ⅰ, (C) Ⅱ, (D) Ⅲ and (E) Ⅳ.

**
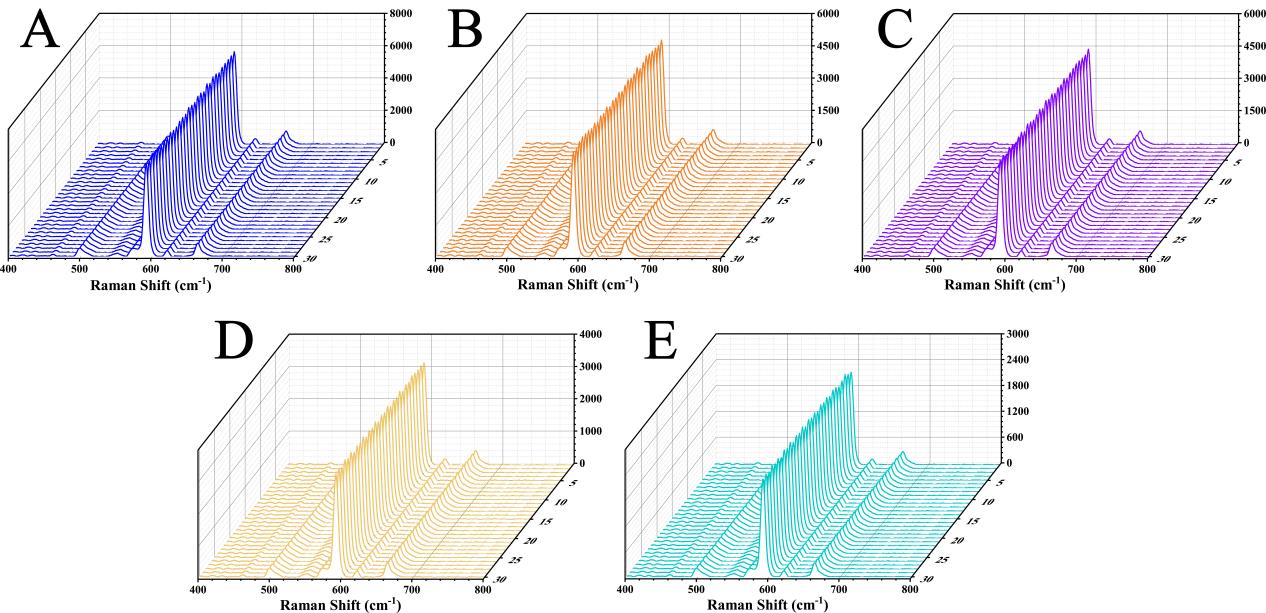
**

**Figure S3** SERS spectra of miR-196b in serum obtained from (A) healthy subjects, (B) LSCC patients at stage (B) Ⅰ, (C) Ⅱ, (D) Ⅲ and (E) Ⅳ.

**Table S1** Result of the SERS-LFA biosensor and qRT-PCR for the real samples (healthy subjects)

|  | SERS (fM) | | qRT-PCR (fM) | | Relative error (%) | |
| --- | --- | --- | --- | --- | --- | --- |
| Sample | miR-106b | miR-196b | miR-106b | miR-196b | miR-106b | miR-196b |
| 1 | 0.194 | 0.143 | 0.186 | 0.138 | 4.071 | 3.426 |
| 2 | 0.199 | 0.132 | 0.191 | 0.124 | 4.029 | 6.438 |
| 3 | 0.189 | 0.140 | 0.203 | 0.132 | -7.597 | 5.915 |
| 4 | 0.191 | 0.141 | 0.179 | 0.137 | 6.182 | 3.071 |
| 5 | 0.191 | 0.148 | 0.183 | 0.153 | 4.165 | -3.248 |
| 6 | 0.173 | 0.156 | 0.164 | 0.151 | 5.324 | 3.107 |
| 7 | 0.160 | 0.138 | 0.149 | 0.129 | 6.786 | 6.477 |
| 8 | 0.203 | 0.155 | 0.192 | 0.147 | 5.242 | 5.481 |
| 9 | 0.193 | 0.132 | 0.199 | 0.127 | -3.096 | 3.496 |
| 10 | 0.188 | 0.135 | 0.179 | 0.130 | 5.049 | 3.503 |
| 11 | 0.173 | 0.152 | 0.162 | 0.147 | 6.296 | 3.152 |
| 12 | 0.171 | 0.140 | 0.165 | 0.135 | 3.551 | 3.604 |
| 13 | 0.169 | 0.147 | 0.157 | 0.153 | 6.878 | -3.775 |
| 14 | 0.182 | 0.143 | 0.173 | 0.135 | 4.808 | 5.905 |
| 15 | 0.195 | 0.149 | 0.181 | 0.141 | 7.249 | 5.421 |
| 16 | 0.158 | 0.149 | 0.151 | 0.139 | 4.226 | 6.902 |
| 17 | 0.167 | 0.147 | 0.157 | 0.141 | 5.994 | 3.918 |
| 18 | 0.173 | 0.133 | 0.167 | 0.125 | 3.467 | 6.258 |
| 19 | 0.166 | 0.142 | 0.155 | 0.133 | 6.894 | 6.356 |
| 20 | 0.160 | 0.144 | 0.149 | 0.139 | 6.748 | 3.288 |
| 21 | 0.159 | 0.152 | 0.146 | 0.144 | 7.867 | 5.312 |
| 22 | 0.203 | 0.153 | 0.214 | 0.146 | -5.253 | 4.621 |
| 23 | 0.180 | 0.150 | 0.172 | 0.158 | 4.536 | -5.193 |
| 24 | 0.175 | 0.147 | 0.166 | 0.140 | 4.883 | 4.742 |
| 25 | 0.201 | 0.146 | 0.193 | 0.141 | 4.025 | 3.491 |
| 26 | 0.171 | 0.138 | 0.164 | 0.132 | 3.833 | 4.166 |
| 27 | 0.201 | 0.142 | 0.191 | 0.134 | 5.052 | 5.446 |
| 28 | 0.188 | 0.150 | 0.177 | 0.143 | 5.743 | 4.418 |
| 29 | 0.195 | 0.136 | 0.186 | 0.142 | 4.838 | -4.418 |
| 30 | 0.160 | 0.142 | 0.154 | 0.132 | 3.792 | 6.742 |

**Table S2** Result of the SERS-LFA biosensor and qRT-PCR for the real samples (stage Ⅰ)

|  | SERS (fM) | | qRT-PCR (fM) | | Relative error (%) | |
| --- | --- | --- | --- | --- | --- | --- |
| Sample | miR-106b | miR-196b | miR-106b | miR-196b | miR-106b | miR-196b |
| 1 | 0.384 | 0.351 | 0.371 | 0.328 | 3.358 | 6.453 |
| 2 | 0.395 | 0.345 | 0.415 | 0.330 | -4.965 | 4.394 |
| 3 | 0.358 | 0.327 | 0.343 | 0.305 | 4.319 | 6.593 |
| 4 | 0.380 | 0.306 | 0.359 | 0.294 | 5.573 | 3.881 |
| 5 | 0.410 | 0.335 | 0.397 | 0.324 | 3.212 | 3.374 |
| 6 | 0.413 | 0.352 | 0.390 | 0.338 | 5.521 | 4.037 |
| 7 | 0.369 | 0.326 | 0.386 | 0.306 | -4.693 | 6.169 |
| 8 | 0.365 | 0.307 | 0.347 | 0.297 | 4.974 | 3.105 |
| 9 | 0.386 | 0.325 | 0.362 | 0.303 | 6.301 | 6.667 |
| 10 | 0.422 | 0.324 | 0.397 | 0.304 | 6.019 | 6.239 |
| 11 | 0.417 | 0.320 | 0.395 | 0.300 | 5.289 | 6.235 |
| 12 | 0.412 | 0.332 | 0.389 | 0.317 | 5.537 | 4.388 |
| 13 | 0.356 | 0.304 | 0.343 | 0.324 | 3.629 | -6.703 |
| 14 | 0.401 | 0.359 | 0.381 | 0.335 | 4.949 | 6.821 |
| 15 | 0.418 | 0.343 | 0.392 | 0.320 | 6.234 | 6.757 |
| 16 | 0.386 | 0.333 | 0.359 | 0.322 | 6.913 | 3.437 |
| 17 | 0.414 | 0.330 | 0.399 | 0.311 | 3.731 | 5.908 |
| 18 | 0.359 | 0.335 | 0.344 | 0.321 | 4.164 | 4.096 |
| 19 | 0.392 | 0.313 | 0.369 | 0.294 | 5.878 | 6.108 |
| 20 | 0.394 | 0.341 | 0.374 | 0.328 | 4.968 | 3.784 |
| 21 | 0.420 | 0.312 | 0.394 | 0.294 | 6.121 | 5.696 |
| 22 | 0.400 | 0.305 | 0.372 | 0.290 | 6.995 | 5.077 |
| 23 | 0.422 | 0.343 | 0.400 | 0.327 | 5.147 | 4.619 |
| 24 | 0.420 | 0.332 | 0.407 | 0.346 | 3.052 | -4.094 |
| 25 | 0.357 | 0.329 | 0.336 | 0.316 | 5.789 | 3.819 |
| 26 | 0.364 | 0.331 | 0.348 | 0.309 | 4.469 | 6.558 |
| 27 | 0.361 | 0.355 | 0.350 | 0.334 | 3.023 | 6.027 |
| 28 | 0.359 | 0.308 | 0.342 | 0.293 | 4.641 | 4.944 |
| 29 | 0.392 | 0.340 | 0.375 | 0.357 | 4.257 | -4.928 |
| 30 | 0.366 | 0.305 | 0.353 | 0.293 | 3.518 | 3.944 |

**Table S3** Result of the SERS-LFA biosensor and qRT-PCR for the real samples (stage Ⅱ)

|  | SERS (fM) | | qRT-PCR (fM) | | Relative error (%) | |
| --- | --- | --- | --- | --- | --- | --- |
| Sample | miR-106b | miR-196b | miR-106b | miR-196b | miR-106b | miR-196b |
| 1 | 0.860 | 0.985 | 0.903 | 0.945 | -5.005 | 4.061 |
| 2 | 0.967 | 1.110 | 0.910 | 1.051 | 5.941 | 5.347 |
| 3 | 0.900 | 1.019 | 0.837 | 0.971 | 6.979 | 4.698 |
| 4 | 0.932 | 1.128 | 0.877 | 1.053 | 5.921 | 6.663 |
| 5 | 0.973 | 1.087 | 0.928 | 1.042 | 4.585 | 4.148 |
| 6 | 0.998 | 1.071 | 0.961 | 1.004 | 3.691 | 6.275 |
| 7 | 0.856 | 1.020 | 0.894 | 0.949 | -4.418 | 6.968 |
| 8 | 0.969 | 1.110 | 0.921 | 1.037 | 4.949 | 6.568 |
| 9 | 0.953 | 1.026 | 0.921 | 0.989 | 3.308 | 3.654 |
| 10 | 0.852 | 0.998 | 0.796 | 0.967 | 6.553 | 3.094 |
| 11 | 0.959 | 1.106 | 0.920 | 1.039 | 4.018 | 6.034 |
| 12 | 0.990 | 1.002 | 0.930 | 0.951 | 6.027 | 5.041 |
| 13 | 0.996 | 1.151 | 0.950 | 1.097 | 4.608 | 4.672 |
| 14 | 0.957 | 1.044 | 0.899 | 1.011 | 6.063 | 3.163 |
| 15 | 0.882 | 1.032 | 0.846 | 0.970 | 4.069 | 5.991 |
| 16 | 0.907 | 1.057 | 0.937 | 0.994 | -3.296 | 5.935 |
| 17 | 0.865 | 1.147 | 0.806 | 1.094 | 6.856 | 4.663 |
| 18 | 0.889 | 1.009 | 0.847 | 0.940 | 4.678 | 6.791 |
| 19 | 0.894 | 1.068 | 0.850 | 1.008 | 4.871 | 5.616 |
| 20 | 0.952 | 1.082 | 0.905 | 1.048 | 4.952 | 3.151 |
| 21 | 0.868 | 1.127 | 0.832 | 1.054 | 4.163 | 6.484 |
| 22 | 0.966 | 1.083 | 0.915 | 1.016 | 5.302 | 6.179 |
| 23 | 0.854 | 1.105 | 0.798 | 1.041 | 6.574 | 5.823 |
| 24 | 0.899 | 1.003 | 0.846 | 0.973 | 5.928 | 3.024 |
| 25 | 0.954 | 0.996 | 0.896 | 0.934 | 6.044 | 6.207 |
| 26 | 0.927 | 1.144 | 0.866 | 1.101 | 6.555 | 3.798 |
| 27 | 0.964 | 1.107 | 0.929 | 1.035 | 3.674 | 6.532 |
| 28 | 0.879 | 1.076 | 0.850 | 1.039 | 3.269 | 3.431 |
| 29 | 0.865 | 1.041 | 0.825 | 0.975 | 4.654 | 6.297 |
| 30 | 0.893 | 1.048 | 0.842 | 0.998 | 5.722 | 4.788 |

**Table S4** Result of the SERS-LFA biosensor and qRT-PCR for the real samples (stage Ⅲ)

|  | SERS (fM) | | qRT-PCR (fM) | | Relative error (%) | |
| --- | --- | --- | --- | --- | --- | --- |
| Sample | miR-106b | miR-196b | miR-106b | miR-196b | miR-106b | miR-196b |
| 1 | 2.293 | 1.365 | 2.164 | 1.273 | 5.618 | 6.731 |
| 2 | 2.053 | 1.315 | 1.918 | 1.275 | 6.564 | 3.012 |
| 3 | 2.049 | 1.422 | 1.987 | 1.360 | 3.048 | 4.386 |
| 4 | 2.212 | 1.410 | 2.067 | 1.352 | 6.541 | 4.141 |
| 5 | 2.297 | 1.360 | 2.205 | 1.300 | 3.998 | 4.428 |
| 6 | 2.056 | 1.438 | 1.932 | 1.339 | 6.052 | 6.875 |
| 7 | 2.279 | 1.361 | 2.187 | 1.302 | 4.016 | 4.314 |
| 8 | 2.227 | 1.474 | 2.123 | 1.416 | 4.655 | 3.965 |
| 9 | 2.137 | 1.463 | 2.022 | 1.396 | 5.371 | 4.597 |
| 10 | 2.144 | 1.377 | 1.989 | 1.335 | 7.227 | 3.065 |
| 11 | 2.301 | 1.334 | 2.194 | 1.258 | 4.652 | 5.695 |
| 12 | 2.289 | 1.388 | 2.199 | 1.328 | 3.927 | 4.317 |
| 13 | 2.311 | 1.347 | 2.196 | 1.282 | 4.987 | 4.832 |
| 14 | 2.088 | 1.394 | 1.937 | 1.319 | 7.216 | 5.366 |
| 15 | 2.273 | 1.354 | 2.188 | 1.307 | 3.754 | 3.504 |
| 16 | 2.150 | 1.351 | 2.043 | 1.298 | 4.972 | 3.916 |
| 17 | 2.007 | 1.411 | 1.878 | 1.361 | 6.422 | 3.539 |
| 18 | 2.331 | 1.337 | 2.249 | 1.271 | 3.525 | 4.954 |
| 19 | 2.121 | 1.469 | 1.990 | 1.409 | 6.155 | 4.079 |
| 20 | 2.237 | 1.369 | 2.075 | 1.282 | 7.254 | 6.342 |
| 21 | 1.990 | 1.439 | 1.892 | 1.366 | 4.912 | 5.085 |
| 22 | 2.307 | 1.395 | 2.199 | 1.318 | 4.675 | 5.532 |
| 23 | 2.169 | 1.407 | 2.040 | 1.360 | 5.944 | 3.317 |
| 24 | 2.335 | 1.397 | 2.251 | 1.334 | 3.606 | 4.515 |
| 25 | 2.141 | 1.314 | 1.971 | 1.251 | 7.962 | 4.784 |
| 26 | 2.191 | 1.368 | 2.072 | 1.323 | 5.416 | 3.309 |
| 27 | 2.143 | 1.423 | 2.028 | 1.363 | 5.382 | 4.192 |
| 28 | 2.223 | 1.454 | 2.091 | 1.370 | 5.958 | 5.76 |
| 29 | 2.009 | 1.476 | 1.872 | 1.424 | 6.827 | 3.512 |
| 30 | 2.072 | 1.429 | 1.973 | 1.335 | 4.773 | 6.569 |

**Table S5** Result of the SERS-LFA biosensor and qRT-PCR for the real samples (stage Ⅳ)

|  | SERS (fM) | | qRT-PCR (fM) | | Relative error (%) | |
| --- | --- | --- | --- | --- | --- | --- |
| Sample | miR-106b | miR-196b | miR-106b | miR-196b | miR-106b | miR-196b |
| 1 | 5.448 | 2.811 | 5.214 | 2.952 | 4.289 | -5.004 |
| 2 | 5.318 | 2.654 | 4.973 | 2.512 | 6.493 | 5.353 |
| 3 | 4.695 | 2.550 | 4.932 | 2.413 | -5.051 | 5.392 |
| 4 | 5.364 | 2.792 | 5.159 | 2.983 | 3.821 | -6.837 |
| 5 | 5.055 | 2.599 | 4.792 | 2.487 | 5.197 | 4.299 |
| 6 | 4.851 | 2.531 | 4.577 | 2.412 | 5.646 | 4.685 |
| 7 | 5.305 | 2.450 | 5.005 | 2.324 | 5.655 | 5.146 |
| 8 | 4.764 | 2.565 | 4.539 | 2.472 | 4.716 | 3.627 |
| 9 | 5.406 | 2.785 | 5.124 | 2.654 | 5.213 | 4.693 |
| 10 | 5.411 | 2.491 | 5.131 | 2.352 | 5.179 | 5.599 |
| 11 | 5.192 | 2.701 | 4.949 | 2.879 | 4.682 | -6.585 |
| 12 | 5.021 | 2.689 | 5.260 | 2.542 | -4.762 | 5.471 |
| 13 | 4.982 | 2.714 | 4.681 | 2.575 | 6.051 | 5.104 |
| 14 | 5.214 | 2.438 | 4.859 | 2.297 | 6.813 | 5.767 |
| 15 | 4.952 | 2.863 | 4.724 | 2.731 | 4.599 | 4.627 |
| 16 | 5.112 | 2.776 | 4.873 | 2.659 | 4.672 | 4.199 |
| 17 | 5.519 | 2.694 | 5.284 | 2.544 | 4.249 | 5.566 |
| 18 | 5.004 | 2.748 | 4.787 | 2.664 | 4.342 | 3.072 |
| 19 | 4.801 | 2.771 | 4.529 | 2.586 | 5.672 | 6.673 |
| 20 | 5.392 | 2.533 | 5.119 | 2.394 | 5.071 | 5.503 |
| 21 | 5.161 | 2.721 | 4.991 | 2.610 | 3.289 | 4.074 |
| 22 | 5.557 | 2.685 | 5.335 | 2.514 | 4.001 | 6.355 |
| 23 | 4.986 | 2.561 | 4.785 | 2.412 | 4.032 | 5.815 |
| 24 | 4.839 | 2.783 | 4.662 | 2.684 | 3.661 | 3.573 |
| 25 | 4.843 | 2.634 | 5.072 | 2.462 | -4.728 | 6.541 |
| 26 | 5.210 | 2.571 | 5.044 | 2.477 | 3.183 | 3.661 |
| 27 | 4.806 | 2.529 | 4.542 | 2.372 | 5.503 | 6.192 |
| 28 | 4.740 | 2.537 | 4.472 | 2.397 | 5.644 | 5.517 |
| 29 | 5.051 | 2.820 | 4.865 | 2.681 | 3.677 | 4.913 |
| 30 | 4.868 | 2.649 | 4.598 | 2.479 | 5.552 | 6.421 |
